# Supplementary material for: Easy-to-actuate multi-compatible truss structures with prescribed reconfiguration
Source: Nat Commun. 2024 Jun 7;15:4886. doi: 10.1038/s41467-024-49210-3 (PMC11161661; doi:10.1038/s41467-024-49210-3)
Supplement: Supplementary file 1 — Supplementary Information [file 41467_2024_49210_MOESM1_ESM.pdf]

Supplementary Information
Easy-to-actuate multi-compatible truss structures with prescribed reconfiguration

Lin Ai[1], Shukun Yin[2], Weixia He[3], Peidong Zhang[1], and Yang Li *[1,4]

[1]The Institute of Technological Sciences, Wuhan University; Wuhan, Hubei, 430072, China
[2]Andrew and Peggy Cherng Department of Medical Engineering, Division of Engineering and Applied Science, California Institute of Technology; Pasadena, California, 91125, United States
[3]Hongyi Honor College, Wuhan University; Wuhan, Hubei, 430072, China
[4]Wuhan University Shenzhen Research Institute, Shenzhen, 518057, China

1 Designing a four-bar linkage passing through n prescribed locations

A single RR dyad consists of a proximal link and distal link, as in Supplementary Fig. 11, denoted as $\mathbf{W_j}$ and $\mathbf{Z_j}$ respectively where the subscript j denoting the configuration numbering. The rotation of the proximal link from the reference (first) configuration is β_j, the rotation of the distal link from the reference configuration in the local coordinate system is θ_j, and the displacement of point $\mathbf{P}$ is $\delta_\mathbf{j}$. The transformation of an RR dyad can be expressed in Euler form as:

$$\delta_\mathbf{j} = \mathbf{P_j} - \mathbf{P_1} = \mathbf{W}\left(e^{i\beta_j} - 1\right) + \mathbf{Z}\left(e^{i\theta_j} - 1\right) \tag{1}$$

where $\delta_\mathbf{j}$ and $\theta_\mathbf{j}$ are prescribed for the jth target configuration, and $\mathbf{W_j}$, $\mathbf{Z_j}$ and β_j are the design variables. The solutions of Eq. (1) are not unique and provide a set of dyads that can achieve the same coupler-link transformation. Designing a dyad going through n prescribed distal-link configurations, the corresponding equations are

$$\begin{cases} \mathbf{P_2} - \mathbf{P_1} = \mathbf{W}\left(e^{i\beta_2} - 1\right) + \mathbf{Z}\left(e^{i\theta_2} - 1\right) \\ \vdots \\ \mathbf{P_j} - \mathbf{P_1} = \mathbf{W}\left(e^{i\beta_j} - 1\right) + \mathbf{Z}\left(e^{i\theta_j} - 1\right) \\ \vdots \\ \mathbf{P_n} - \mathbf{P_1} = \mathbf{W}\left(e^{i\beta_n} - 1\right) + \mathbf{Z}\left(e^{i\theta_n} - 1\right) \end{cases} \tag{2}$$

A four-bar linkage can be constructed by joining two different dyads, which have the same distal-link transformations through satisfying Eq.(2), and the two respective distal links are rigidly connected to form the coupler link with the reference point $\mathbf{P}$.

*Corresponding author: yang.li@whu.edu.cn

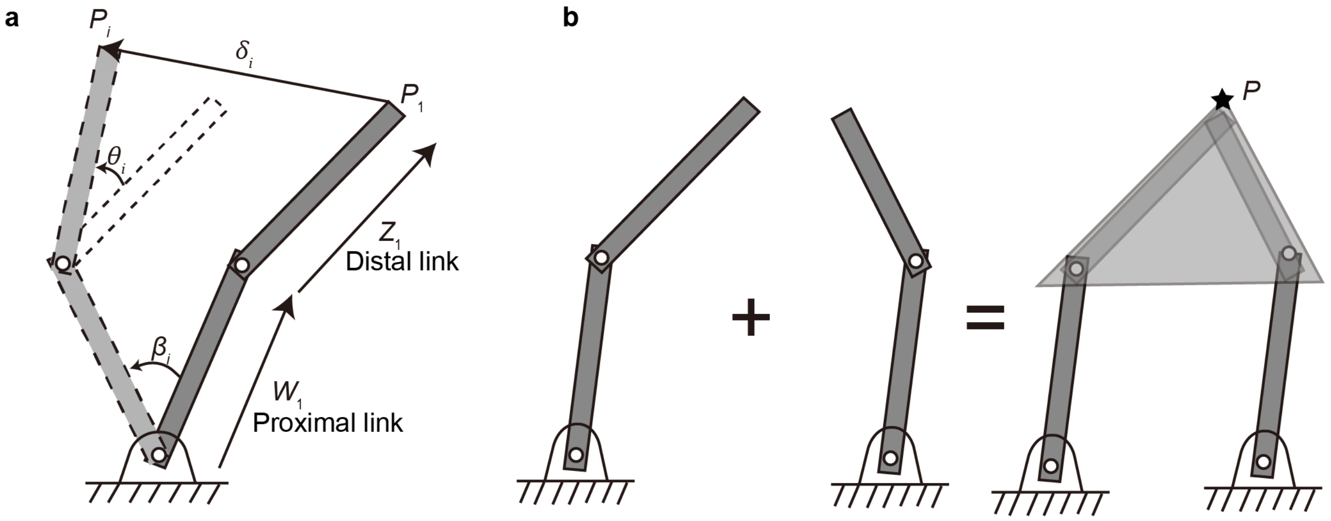

Supplementary Fig. 1. **The rotation of an RR dyad and the formation of a four-bar linkage through jointing two different dyads. a** $\boldsymbol{W}_1$ and $\boldsymbol{Z}_1$ are the proximal link and distal link respectively. β_i and θ_i are the rotation angles of $\boldsymbol{W}_1$ and $\boldsymbol{Z}_1$ from the first configuration to the *i* th configuration. δ_i is the displacement of point ***P*** from the first configuration to the *i* th configuration. **b** A four-bar linkage can be constructed by joining two different dyads, which have the same distal-link transformations, and the two respective distal links are rigidly connected to form the coupler link with the reference point ***P***.

2 General description of multi-compatibility method

Consider a three-dimensional structure with n_c target stable configurations consisting of n_b components, connected by n_r revolute joints, n_u universal joints and n_s spherical joints. Axis-angle representation is employed for a simpler correlation with the physical joint rotation as shown in Supplementary Fig. 2A, in which the joint is represented by a directional vector $\mathbf{k}$ of the rotation axis and a rotation angle θ around the axis. For the universal joints employed, the angle α in between the two directional vectors $\mathbf{k}$ of two axes can be designed instead of being always perpendicular as in the standard universal joints as shown in Supplementary Fig. 2A. The transformation from the axis-angle representation to rotation matrix $\mathbf{R}$ is given in Eq. (3)-(5).

$$\mathbf{k} = (k_x, k_y, k_z) \tag{3}$$

$$\mathbf{K} = \begin{bmatrix} 0 & -k_z & k_y \\ k_z & 0 & -k_x \\ -k_y & k_x & 0 \end{bmatrix} \tag{4}$$

$$\mathbf{R} = \mathbf{I} + (\sin\theta)\mathbf{K} + (1-\cos\theta)\mathbf{K}^2 \tag{5}$$

The prescribed configurations are defined by a set of reference points based on their nodal coordinates and rotation angles from the reference (first) configuration. For example, component p in configuration i is represented by a reference point $\mathbf{P}_i^p$ and a rotation angle $\theta_{\mathbf{i}}^{\mathbf{p}}$ of the component p from the reference configuration in the local coordinate system. Matrix $\mathbf{P}$ includes coordinates of all reference points, and matrix θ contains all rotation angles as shown in Eq. (6). Thus, these two matrices are prescribed input to the design problem as the target of designing a multi-stable structure.

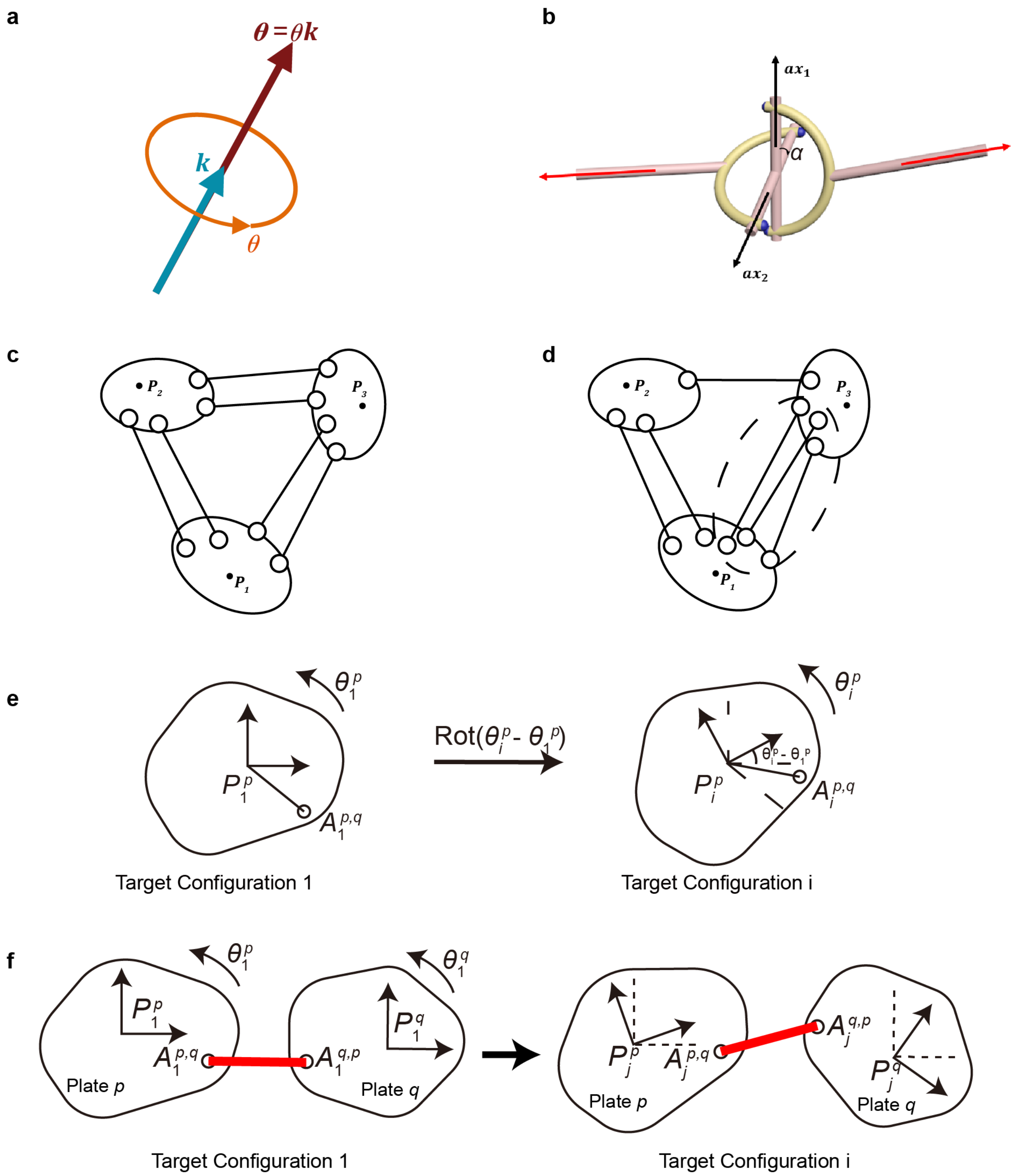

Supplementary Fig. 2. **The general multi-stable structure design method is based on multi-compatibility. a** The variables of the axis-angle representation of a rotation: the directional vector of the rotation axis $\boldsymbol{k}$, the rotation angle θ. **b** The schematic diagram of the universal joint. The angle α in between the two axes $\boldsymbol{ax}_1$ and $\boldsymbol{ax}_2$ could be designed rather than to be perpendicular. **c** One example of the topology that illustrates the kinematic DOF of the whole system should be no bigger than 0. **d** Another suggestion to the topology: the kinematic DOF of subsystems should be bigger than 0. **e** The rotation of each component from the first configuration to the i th configuration. **f** The multi-compatibility constraint: different components rotate to different angles, and the coordinates of the joint connected to them should be identical after the transformation.

$$\mathbf{P} = \begin{bmatrix} \mathbf{P_1^1} \\ \vdots \\ \mathbf{P_i^p} \\ \vdots \\ \mathbf{P_{n_c}^{n_b}} \end{bmatrix}, \quad \theta = \begin{bmatrix} \theta_\mathbf{1}^\mathbf{1} \\ \vdots \\ \theta_\mathbf{i}^\mathbf{p} \\ \vdots \\ \theta_\mathbf{n_c}^\mathbf{n_b} \end{bmatrix} \tag{6}$$

To design a truss-like structure (as Supplementary Fig. 2C) to be correspondingly multi-compatible, three types of variables need to be considered: (1) topological variables controlling which components are connected with what type of joints. Matrix $\mathbf{T}$ are topology variables geometry, assigning the DOF of the joint connecting body p and body q as $\mathbf{T}^{p,q}$; (2) geometrical variables determining the shape of bodies. Matrix $\mathbf{A}$ assembles the geometry variables assigning the coordinates of the joint connecting body p and body q in configuration i as $\mathbf{A_i^{p,q}}$; and (3) kinematic variables are used to determine directional vectors and rotation angles about different axes in a joint. We use matrix Θ to represent joint variables, assigning the rotation vectors of different axes in the joint connecting body p and body q in configuration i as $\Theta_i^{p,q}$. When all components are prescribed target configurations in Eq. (6), then the kinematic variable Θ equals to θ. When some components are relaxed from the exact prescribed targets, the reference points and rotation angles of these components are kinematic variables to be solved. The above three types of variables are called design variables, which determine the appearance of multi-stable structures. Then the formulations of the above parameters are introduced.

Topology not only determines how bodies connect to each other, but also determines the DOF of the whole or partial system of the structure. When all components are considered rigid, topological variables matrix $\mathbf{T}$ should satisfy the following conditions for the system to be a multi-stable structure:

Condition 1 The kinematic DOF of the whole system should be no bigger than 0.

Condition 2 The kinematic DOF of subsystems should be bigger than 0.

The first condition ensures the system becomes an immovable structure, rather than a mechanism whose energy always equals zero. When the number of DOF is negative, the system becomes a statically indeterminate structure, where the number of undesigned stable states may be reduced since there are more constraints, which is discussed in Section 10.2. The second condition is also necessary to make sure there is no local rigidity in order to avoid redundant stable states appearing in the subsystems. These two conditions make sure the structure is closed-loop and avoid any open-loop or partial open-loop situation. The schematic diagrams of two conditions are plotted in 2D as Supplementary Fig. 2C and Supplementary Fig. 2D, noting that a component is simplified into a bar with two revolute joints in 2D. Based on the C-G-K criterion, these conditions are formulated as Eqs. (7)-(8):

$$M_w = 6\left(n_b - 1\right) - \sum_{p=1, p<q}^{n_b} \sum_{q=2}^{n_b} \left(6 - T^{p,q}\right) \leq 0 \quad \text{for } p, q = 1, ..., n_b \tag{7}$$

$$M_p = 6\left(n_b' - 1\right) - \sum_{p', p'<q'}^{n_b'} \sum_{q'}^{n_b'} \left(6 - T^{p',q'}\right) > 0 \quad \text{for } p', q' \in C \tag{8}$$

where M_w stands for kinematic DOF of the whole system, and M_p for kinematic DOF of subsystems. p' and q' are in set C, where C is a subset of all components with the number of elements in the range of 2 to $n_b - 1$. n_b' refers to the number of elements in set C.

The above DOF calculation has designed the DOF of each joint in the structure, but the design parameters of the joints have not been calculated, such as the angle α between two axes in a universal joint (shown as Supplementary Fig. 2). Regardless of the DOF of the joint, obtain the rotation (denoted by the axis-angle representation) incurred by this joint through the following geometry design. Finally, design the joints satisfying both the above DOF and rotation relationship.

Geometric variables $\mathbf{A}$ are designable, consisting of only the coordinates of all joints at the first configuration, parameterized as

$$\mathbf{A} = \mathbf{A}_1 = \begin{bmatrix} \mathbf{A}_1^1 \\ \vdots \\ \mathbf{A}_1^p \\ \vdots \\ \mathbf{A}_1^{n_b} \end{bmatrix} \tag{9}$$

where $\mathbf{A}_1^p \in \Re^{dq_p}$ is a vector of coordinates of joints on the p th body, q_p means the number of joints on this body, d could be 2 or 3 depending on the dimension in which this design problem would be discussed, and $\mathbf{A} \in \Re^{dn_b n_b}$ is a matrix of all coordinates of joints at the first configurations.

According to the prescribed input $\mathbf{P}$ and θ as shown in Eq. (6), the coordinates of all joints at ith target configurations ($i > 1$) can be expressed by Eq. (9). Supplementary Fig. 2E presents a top view of one component transforming from configuration *1* to configuration *i*. The rotation equations are written to describe its transformations:

$$\mathbf{A}_i^{p,q} - \mathbf{P}_i^p = Rot(\theta_\mathbf{i}^\mathbf{p}) \cdot (\mathbf{A}_1^{p,q} - \mathbf{P}_1^p) \tag{10}$$

where the subscript i represents the number of target configurations while the superscript p,q represents which two bodies are connected at this joint. The first superscript represents the coordinates of this point A which are derived from the rotation of the pth body. The function $Rot(\theta)$ would be different depending on the dimension of the problem to be solved.

Geometric variables are to be solved in a constrained optimization framework that satisfies the "multi-compatibility" constraint. Multi-compatibility means all components in the structure have no deformation at multiple prescribed target configurations so that total strain energy equals zero. Thus, the structure is globally multi-stable. Eq. (10) has ensured each component is multi-compatible at multiple prescribed configurations and prevents components from symmetrically flipping. In addition, the coordinates of all joints at other configurations are derived from the reference configuration. This parameterization can decrease the number of constraints as well as the number of variables. Therefore, for the constrained optimization solver, the reduction of the number of constraint equations may increase the probability of successful convergence.

Each component suffers from different prescribed motions, such as body p moves from $\mathbf{P}_i^p$ to $\mathbf{P}_{i+1}^p$ and rotate an angle θ_{i+1}^p while body q moves from $\mathbf{P}_i^q$ to $\mathbf{P}_{i+1}^q$ and rotate an angle θ_{i+1}^q, thus the coordinates of point $\mathbf{A}_{i+1}^{p,q}$ and point $\mathbf{A}_{i+1}^{q,p}$ would be different. However, these two points are connected by one joint physically, so another condition is proposed to ensure the coordinates of certain nodes should be identical if these nodes are connected by the same joint, as fig. S4F, which is equivalent to stating:

$$|\mathbf{A}_i^{p,q} - \mathbf{A}_i^{q,p}| = 0 \quad for\ i = 1, 2, ..., n_c \tag{11}$$

In addition, if the right-hand side of Eq. (11) is greater than zero, it means there is a bar connected between these two components.

In addition to designing a multi-stable structure that can achieve multiple prescribed configurations with the multi-compatibility equality constraints discussed above, other inequality constraints could be added to make the structure neater. For example, the size of each component could be constrained to a certain range, including the distance between the reference points and joints (as Eq. (12)) and the distance among joints (as Eq. (13)):

$$lb \leq \left|\mathbf{P}_1^p - \mathbf{A}_1^{p,Q_p(q)}\right| \leq ub \quad for\ p = 1, ..., n_b,\ q = 1, ..., q_p \tag{12}$$

$$lb \leq \left|\mathbf{A}_1^{p,Q_p(i)} - \mathbf{A}_1^{p,Q_p(j)}\right| \leq ub \quad for\ p = 1, ..., nb,\ i = 1, ..., (q_p - 1),\ j = (i+1), ..., q_p \tag{13}$$

where p and q index the number of bodies, Q_p records all the serial numbers of the bodies that are connected with the body p, q_p denotes the number of bodies that are connected to the body p, lb means the lower boundary of the sizes of bodies and ub means the upper boundary of the sizes of bodies, lb and ub in the Eq. (12)-(13) could be set different values. The setting of these boundaries can refer to the optimization results only with the necessary multi-compatibility constraints.

Then designing a multi-compatible structure achieving multiple prescribed configurations can be framed as a constrained optimization problem:

$$\underset{\mathbf{A}}{min} E(\mathbf{A}) \quad subject\ to \quad Ceq_{cpt}(\mathbf{A}),\ C_{size}(\mathbf{A}) \tag{14}$$

where the expression of the cost function E depends on the specific application. A fundamental cost function is the sum of bodies' size considering the cost of fabrication,

$$E(\mathbf{A}) = \sum_{p=1,...,n_b}^{q=1,...,q_p} |\mathbf{P}_1^p - \mathbf{A}_1^{p,Q_p(q)}| + \sum_{p=1,...,n_b} \sum_{j=(i+1),...,q_p}^{i=1,...,(q_p-1)} |\mathbf{A}_1^{p,Q_p(i)} - \mathbf{A}_1^{p,Q_p(j)}| \tag{15}$$

The constraints are divided into two sets: $Ceq_{cpt}(\mathbf{A}),\ C_{size}(\mathbf{A})$. The first set of constraints, $Ceq_{cpt}(\mathbf{A})$, defines the structure as multi-compatible at several prescribed configurations simultaneously:

$$Ceq_{cpt}(\mathbf{A}) = \left\{ |\mathbf{A}_i^{p,Q_p(q)} - \mathbf{A}_i^{Q_p(q),p}| = 0, \qquad for\ i = 1, , ..., n_c,\ p = 1, .., n_p, q = 1, ..., q_p \right\} \tag{16}$$

The second set of constraints, $C_{size}(\boldsymbol{A})$, constrained the size of the structure to render it neater,

$$C_{size}(\mathbf{A}) = \left\{ \begin{array}{c} lb \leq \left| \mathbf{P}_1^p - \mathbf{A}_1^{p,Q_p(q)} \right| \leq ub, \quad for\ i = 1, , ..., n_c,\ p = 1, .., n_p, q = 1, ..., q_p \\ lb \leq \left| \mathbf{A}_1^{p,Q_p(i)} - \mathbf{A}_1^{p,Q_p(j)} \right| \leq ub, \quad for\ p = 1, .., n_p, i = 1, , ..., (q_p - 1),\ j = (i+1), ..., q_p \end{array} \right\} \tag{17}$$

In order to design a multi-compatible structure that precisely achieves target configurations, the coordinates and rotations of reference points at all components are specified. However, according to Section 10.2, prescribing all reference points and rotations is too ideal to overconstrain the optimization. In such cases, it is of interest to relax the constraints of fitting targets to obtain feasible solutions. The relaxation of the design problem means prescribing the exact values of reference point coordinates and rotation angles only on selected components depending on applications, and the reference points and rotations of other components are set as variables to be optimized by minimizing the cost function, describing deviation between the actual configurations and prescribed target configurations:

$$E = E_{size} + w_1 E_{d_{Pos}} + w_2 E_{d_{Ang}} \tag{18}$$

where w_1 and w_2 are the weight of terms, the first term E_{size} in the cost function is the sum of all components' sizes as Eq. (15). The latter two terms penalize the deviation of the reference points' positions ($E_{d_{Pos}}$) and rotation angles ($E_{d_{Ang]}}$), which are defined as:

$$E_{d_{Pos}} = \sum_{i=1,...,n_c}^{p=1,...,n_p} |\mathbf{P}_{act}{}_i^p - \mathbf{P}_{tar}{}_i^p| \tag{19}$$

$$E_{d_{Ang}} = \sum_{i=1,...,n_c}^{p=1,...,n_p} |\theta_{act}{}_i^p - \theta_{tar}{}_i^p| \tag{20}$$

where the subscript act means the actual reference points or rotation angles in this structure while the subscript tar means the target ones that we prescribe. It is noted that $\mathbf{P}$ and θ with the subscript act are variables to be optimized while $\mathbf{P}$ and θ with the subscript tar are prescribed.

3 Local stiffness characterization

The local stiffness is a significant property of the multi-stable structure. The bar and hinge approach is used to model the structure with elastic behavior and the eigenvalues of the stiffness matrix are calculated to explore the stiffness characteristics [1]. The plate in 2D is highly resistant to buckling so it can be triangulated to remain planar. Similarly, the body in 3D can be modeled by tetrahedron to ensure the body may not deform. Then the constituents of the structure are pin-jointed bars, as shown in Supplementary Fig. 3A and S3B.

3.1 Compatibility matrix and equilibrium matrix

Consider a structure consisting of n_b bars connected by n nodes. The coordinates of the initial configuration are $\mathbf{x^0}$, and the coordinates of the current configuration are $\mathbf{x^i}$, having experienced i prediction steps. The exact geometry difference between the coordinates $\mathbf{x^0}$ and $\mathbf{x^i}$ represents the deformation. Specifically, the change of distance between two nodes (and the variation of fold angle between two triangles) is the geometry difference. For pin-jointed bars, the lengths of all bars with $\mathbf{x^0}$ can be computed using the distance formula and are considered to be the original bar lengths. Similarly, the lengths of the bars with $\mathbf{x^i}$ can be achieved, and their differences from the original bar lengths constitute the deformation, which is denoted e_b^i. Then the relationship between the deformation of bars e_b^i and the displacement of nodes d^i could be derived. Considering a general k bar that connects node i to node j as Supplementary Fig. 3C, a small displacement $[u_k^i v_k^i u_k^j v_k^j]$ is applied to nodes. This nodal displacement provides the bar's extension e_b^k, which could be expressed as

$$-cos\alpha_k u_k^i - sin\alpha_k v_k^i + cos\alpha_k u_k^j + sin\alpha_k v_k^j = e_b^k \tag{21}$$

because $sin\alpha_k$ and $cos\alpha_k$ could be expressed by the coordinates of nodes i and j, above equation can be rewritten as

$$\frac{x_i - x_j}{L_k} u_k^i + \frac{y_i - y_j}{L_k} v_k^i + \frac{x_j - x_i}{L_k} u_k^j + \frac{y_j - y_i}{L_k} v_k^j = e_b^k \tag{22}$$

which can be easily extended to 3 dimensions. The above equation is called the compatibility equation, which provides a relationship between first-order deformation and nodal displacements. Putting together the compatibility equations of all bars, they can be written in a matrix form as

$$\mathbf{C}_b \mathbf{d} = \mathbf{e}_b \tag{23}$$

where $\mathbf{C}_b$ is a $n_b \times 3n$ matrix, called compatibility matrix, $\mathbf{d}$ is a column vector with $3n$ components, $\mathbf{e}^b$ is the extension vector of bars with n_b components [2].

The stress of bars could be obtained through the deformation of bars. The equilibrium equation describes the relationship between the stress of bars and the load at nodes, derived as follows: take joint h as an example of equilibrium equations as Supplementary Fig. 3D, a load $\mathbf{p}$ is applied to the joint h, connected by bar f to joint i and by bar g to joint j:

$$\begin{cases} -cos\alpha_f t_f - cos\alpha_g t_g = p_{hX} \\ -sin\alpha_f t_f - sin\alpha_g t_g = p_{hY} \end{cases}. \tag{24}$$

Use the coordinates of nodes i and h to express $sin\alpha_f$, $cos\alpha_f$, $sin\alpha_g$, and $cos\alpha_g$ as:

$$sin\alpha_f = \frac{Y_i - Y_h}{L_f} \quad and \quad cos\alpha_f = \frac{X_i - X_h}{L_f},\ etc \tag{25}$$

where L_f is the length of bar f. Substituting these expressions into Eq. (26) gives:

$$\begin{cases} \frac{X_h - X_i}{L_f} t_f - \frac{X_h - X_j}{L_g} t_g = p_{hX} \\ \frac{Y_h - Y_i}{L_f} t_f - \frac{Y_h - Y_j}{L_g} t_g = p_{hY} \end{cases}. \tag{26}$$

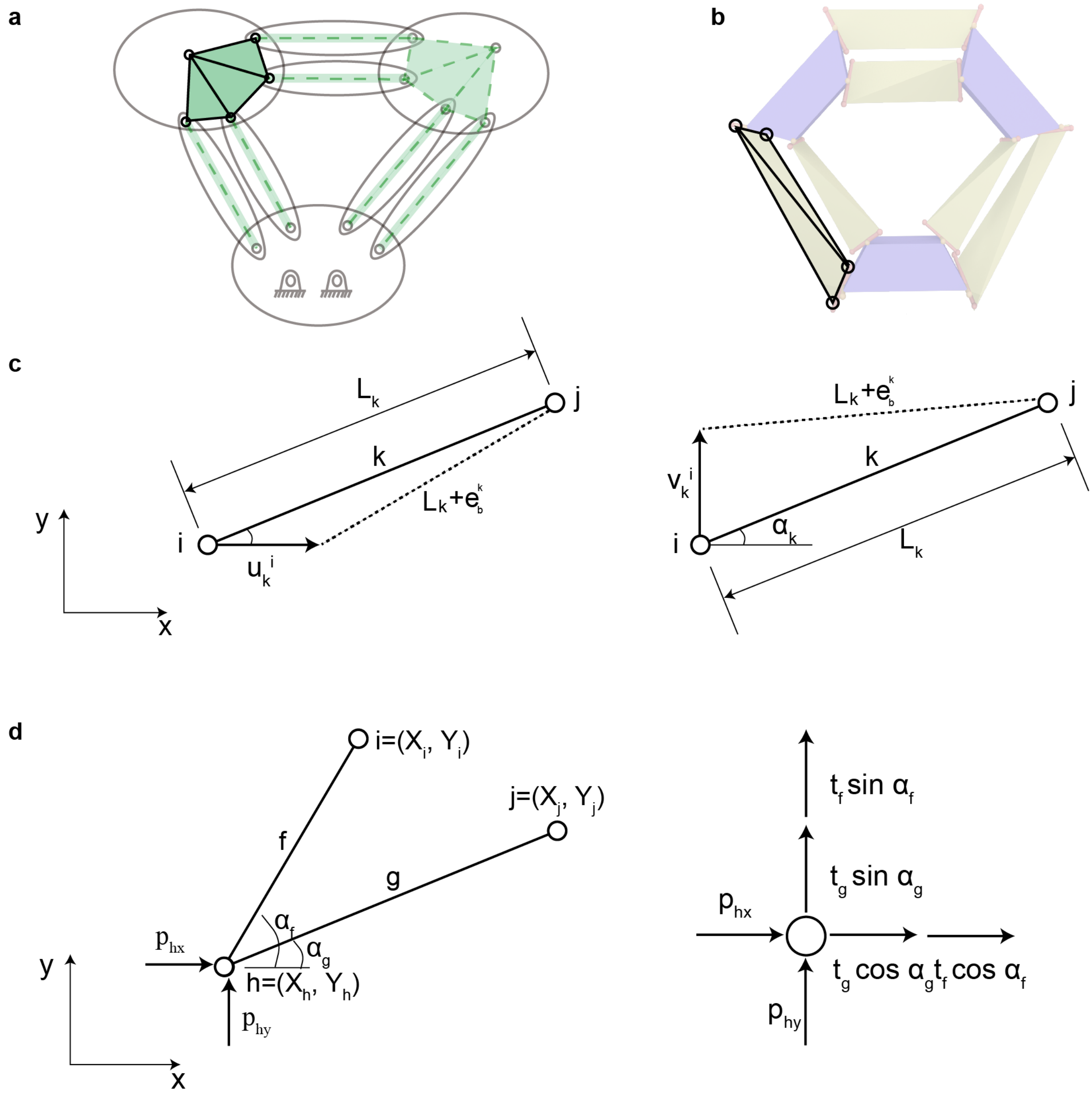

Supplementary Fig. 3. **The simulation model and formation of the compatibility matrix.** **a** The two-dimensional bar-and-plate structure is modeled in terms of pin-jointed bars, and plates are represented by connecting pin-jointed bars into triangles. **b** The three-dimensional bar-and-body structure is connected by universal joints, which could form any angle. The bars with universal joints are modeled in terms of pin-jointed bars to form tetrahedrons, and the bodies are also subdivided into tetrahedrons. **c** Formation of the compatibility matrix. A small displacement is applied to nodes i, considering a general bar k that connects node i to node j. This nodal displacement provides the bar's extension e_b^k. **d** Formation of the equilibrium matrix. A load p is applied to the joint h, connected by bar f to joint i and by bar g to joint j and then the force reaches equilibrium at the joint h.

Putting together the equilibrium equations of all nodes could derive the equilibrium matrix,

$$\mathbf{Ht} = \mathbf{p}. \tag{27}$$

The equilibrium matrix $\mathbf{H}$ is the transpose of the compatibility matrix $\mathbf{C}$.

3.2 Eigen decomposition of stiffness matrix

The key idea of the bar and hinge model is to follow this natural discretization as well as to provide bar elements with axial stiffness, that a value of 1000 for rigid bars forming the triangles in plates or tetrahedron in bodies, while the value of 1 for elastic bars. Then the stress of elements could be calculated through the strain:

$$\mathbf{D}_k\mathbf{e} = \mathbf{t} \tag{28}$$

where $\mathbf{D}_K$ shows the stiffness of all elements in the form of a diagonal matrix, $\mathbf{e}$ represents the strain of elements, $\mathbf{t}$ means the stress in the elements. Introduce the compatibility matrix $\mathbf{C}$ relating the nodal displacements to the generalized strain, and the equilibrium matrix $\mathbf{H}$ relating the generalized stress to the load:

$$\mathbf{Ht} = \mathbf{p}, \quad \mathbf{Cd} = \mathbf{e} \tag{29}$$

where $\mathbf{t}$ means the stress in the elements, $\mathbf{p}$ denotes the load at nodes, $\mathbf{d}$ denotes the displacement of nodes, and $\mathbf{e}$ represents the strain of elements.

Then the stiffness matrix of nodes is assembled from Eq. (28), (29) as follows:

$$\mathbf{K} = \mathbf{HD}_K\mathbf{C}. \tag{30}$$

It is possible to analyze the local stiffness by obtaining the eigenvalues λ_i and the eigenmodes $\mathbf{v}_i$ of the stiffness matrix as:

$$\mathbf{Kv}_i = \lambda_i\mathbf{v}_i. \tag{31}$$

The eigenvalues are arranged in incremental order and represent the elastic energy that is induced by moving along the corresponding eigenmode. The lowest elastic energy corresponds to the most flexible eigenmode, along which the structure transforms from the current configuration the most easily. So we used the first eigenvalue to characterize the local stiffness of the multi-stable structures at this configuration. Here we compare the local stiffness between a designed 2P4B structure based on multi-compatibility in Fig. 2A with another tri-stable structure, which consists of a simple mechanism (four-bar-linkage) with elastic components (linear springs) and is designed from an energy perspective [3], as shown in Supplementary Fig. 4. These two methods both conduct an inverse design of a tri-stable structure and no need for complex actuation, although the description of prescribed configurations is different. We can see that the stability of our multi-stable structure (Supplementary Fig. 4A) is higher than the other one (Supplementary Fig. 4B) generally, but the transformation path between stable states is not given by the structure itself while the other method could be because the base of the structure, a simple mechanism, decides the transformation path.

Furthermore, the energy barrier between stable states could be influenced by the local stiffness design which specifies the local curvature of the energy landscape. If the local stiffness of the two configurations are both high, then the energy barrier of the transformation between them would be high. If both of their local stiffness are low, then the energy barrier would be low. If only one of them is high, then the energy barrier would be of medium height. Then, different configurations with different stability to achieve high energy efficiency could be designed. A low energy barrier means the structure consumes less energy to be actuated to the next configuration, while a high energy barrier means the structure could maintain a configuration more firmly without energy consumption. However, with the complexity of the topology of multi-stable structures increasing, the nonlinearity of the motion of each

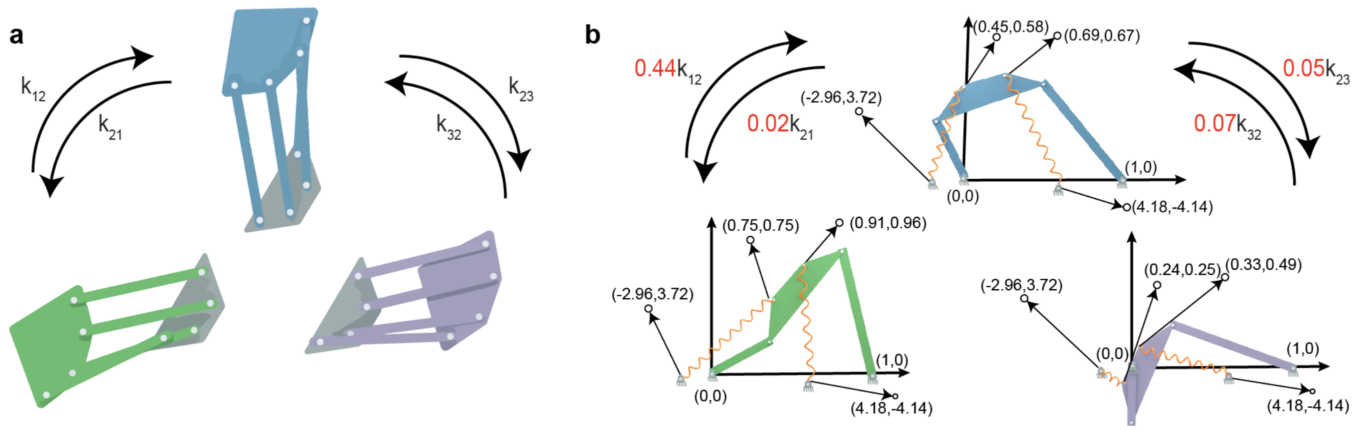

Supplementary Fig. 4. **The local stiffness characterization and comparison between two tri-stable structures designed by different methods. a** Local stiffness of a tri-stable structure designed by the multi-compatibility method proposed in this paper. The local stiffness is expressed as the ratio of the smallest eigenvalue of the stiffness matrix to the stiffness of bars. k_{12}=0.0120/1, k_{23}=1.7439/1, k_{32}=0.2153/1, k_{21}=0.0083/1. **b** Local stiffness of a tri-stable structure consisting of rigid mechanism (four-bar-linkage) and elastic elements (linear springs) designed from energy perspective (Y. Li and Pellegrino 2020). The stability of the tri-stable structure designed in this paper is higher than the other generally.

component is more prominent, and the relationship between local stiffness and energy barrier becomes vaguer, so it is hard to predict the energy barrier through values of the local stiffness for complex multi-stable structures.

4 Minimum energy path (MEP) finding

4.1 Axial load versus axial displacement relationship of different elements

The axial load versus axial displacement relationship is different for different elements. Here we discussed the truss element and the beam element. Truss elements are simple two-node linear members that only take axial extension or compression. The stress of the elements can be defined as:

$$t = k_t x \tag{32}$$

where k is the stiffness of the element, x is the axial displacement of the node. Then the nodal load can be obtained by projecting the stress by the equilibrium matrix in Supplementary Equation (29).

When the beam element is subjected to compression, it appears to bend. When the beam is subjected to tension, the beam cannot be stretched, which is approximated by a bar with high stiffness. The stress of beam elements described by the axial load versus axial displacement relationship can be formulated as:

$$t = \begin{cases} -P_{cr}\left(1 - \frac{x}{2l}\right), & x < 0 \\ k_b x, & x \geq 0 \end{cases} \tag{33}$$

where l is the original length of the element, k_b is set to 10^4 times the slope of the bending force formula, P_{cr} is the critical load, written in the form

$$P_{cr} = \frac{\pi^2}{l^2} EI \tag{34}$$

where E is Young's modulus, I is the moment of inertia. To avoid the sudden change of the stress in the beam when the axial displacement changes the sign, the piecewise function in Supplementary Equation (33) needs to be smoothly blended.

4.2 Nudged Elastic Band (NEB)

The nudged elastic band (NEB) method [4] is widely used in computational chemistry to calculate transition states in chemical systems. Furthermore, it is utilized to identify rigid and deformable folding paths in origami design [5]. Fig. 1C shows the principle of the NEB method. $\mathbf{C_1}$ and $\mathbf{C_N}$ are two target stable states that are obtained from the multi-compatibility design. We firstly interpolate linearly $(N-2)$ points between $\mathbf{C}_1$ and $\mathbf{C}_N$, $\mathbf{C}_2, ..., \mathbf{C}_{(k-1)}, \mathbf{C}_k, \mathbf{C}_{(k+1)}, ..., \mathbf{C}_{(N-1)}$, each point represents an intermediate configuration during the transformation. The elastic energy of each configuration is expressed as $E_e(\mathbf{C}_k)$, that is, the sum of the strain energy of all components. The strain energy of each component is obtained by integrating the corresponding force vs. displacement function in Supplementary Equation (32) and (33). Then the overall strain energy is the sum of that at all configurations:

$$E_e^{tol} = \sum_{k=1}^{N} E_e(\mathbf{C}_k). \tag{35}$$

We aim to explore a transformation path with minimum energy connecting two stable states, which means the energy would increase in any direction except the tangent direction of the path at each configuration. Moreover, in order to avoid the path just connecting local energy minimum roughly without the continuity of a path, assume connecting some linear springs between the configurations along the path to be an elastic band, and minimize the elastic energy of the band $E_b(\mathbf{C}_k)$ to ensure a smooth path.

$$E_b(\mathbf{C}_k) = \frac{1}{2} k_b \left|\mathbf{C}_k - \mathbf{C}_{k-1}\right|^2 \tag{36}$$

where k_b is the elastic constant of springs between states. So the overall energy of the elastic band is as:

$$E_b^{tol} = \sum_{k=2}^{N} E_b(\mathbf{C}_k). \tag{37}$$

Then the negative gradients of the two types of respective energy are:

$$\sigma_k^e = -\nabla E_e(\mathbf{C}_k), \quad \sigma_k^b = k_b(|\mathbf{C}_{k+1} - \mathbf{C}_k| - |\mathbf{C}_k - \mathbf{C}_{k-1}|). \tag{38}$$

However, simply moving along the sum of $\sigma_e(C_k)$ and $\sigma_b(C_k)$ may cause the distribution of intermediate configuration points to be sparse at peaks of the energy landscape and dense at valleys. This is due to the additional tension in the band at the concave landscape while compression at convex locations. To resolve this problem, σ_k^e is projected into the normal direction of the path as $\sigma_k^{e,\perp}$ and project the spring energy term σ_k^b tangential to the path $\sigma_k^{b,\parallel}$, the superscripts $\perp$ and $\parallel$ represent the perpendicular and parallel components with respect to the tangent of the path τ_k respectively, so the interaction between $\sigma_e(C_k)$ and $\sigma_b(C_k)$ would be eliminated, and a minimum energy path (MEP) with evenly spaced intermediate configuration points can be found when iteratively moving along the negative energy gradient

$$\sigma_k = \sigma_k^{e,\perp} + \sigma_k^{b,\parallel} \tag{39}$$

The precise calculation for the tangent of the path τ_k is defined as forward, backward, or central difference schemes according to different conditions of physical strain energy term variations [5]:

$$\tau_k = \mathbf{u}_k / |\mathbf{u}_k| \tag{40}$$

$$\mathbf{u}_k = \begin{cases} \mathbf{C}_{k+1} - \mathbf{C}_k, \quad if\ E_e(\mathbf{C}_{k+1}) \geq E_e(\mathbf{C}_k) \geq E_e(\mathbf{C}_{k-1}) \\ \mathbf{C}_k - \mathbf{C}_{k-1}, \quad if\ E_e(\mathbf{C}_{k+1}) \leq E_e(\mathbf{C}_k) \leq E_e(\mathbf{C}_{k-1}) \\ (\mathbf{C}_{k+1} - \mathbf{C}_k) \triangle E_{e_k}^{max} + (\mathbf{C}_k - \mathbf{C}_{k-1}) \triangle E_{e_k}^{min}, \quad if\ E_e(\mathbf{C}_k) extrema,\ E_e(\mathbf{C}_{k+1}) > E_e(\mathbf{C}_{k-1}) \\ (\mathbf{C}_{k+1} - \mathbf{C}_k) \triangle E_{e_k}^{min} + (\mathbf{C}_k - \mathbf{C}_{k-1}) \triangle E_{e_k}^{max}, \quad if\ E_e(\mathbf{C}_k) extrema,\ E_e(\mathbf{C}_{k+1}) \leq E_e(\mathbf{C}_{k-1}) \end{cases} \tag{41}$$

where

$$\triangle E_{e_k}^{max} = max(|E_e(\mathbf{C}_{k+1}) - E_e(\mathbf{C}_k)|, |E_e(\mathbf{C}_k) - E_e(\mathbf{C}_{k-1})|) \tag{42}$$

$$\triangle E_{e_k}^{min} = min(|E_e(\mathbf{C}_{k+1}) - E_e(\mathbf{C}_k)|, |E_e(\mathbf{C}_k) - E_e(\mathbf{C}_{k-1})|) \tag{43}$$

Then in order to find the optimal path, the descent-type algorithm is utilized. The minimization algorithm is terminated when two gradient terms $\sigma_k^{e,\perp}$ and $\sigma_k^{b,\parallel}$ converge. The details of the optimization procedure are summarized in Algorithm 1.

4.3 Comparison of MEP obtained by the beam model and truss model

For the 2P4B structure, we applied the beam model in the NEB method and compared the MEP obtained by the truss model and beam model as shown in Supplementary Fig. 5. Although the energy curves, i.e. energy barrier, are a little different, the deformed configurations between the stable states are identical. Besides, the convergence of the NEB by the beam model took longer than that by the truss model. For the actuation design, it is significant to obtain the middle configurations during the reconfiguration rather than calculate accurate energy values. Therefore, if the values of energy barriers are important, the beam model can be chosen, if only requires designing the actuation at a lower time cost, it would be better to apply the truss model in the MEP finding.

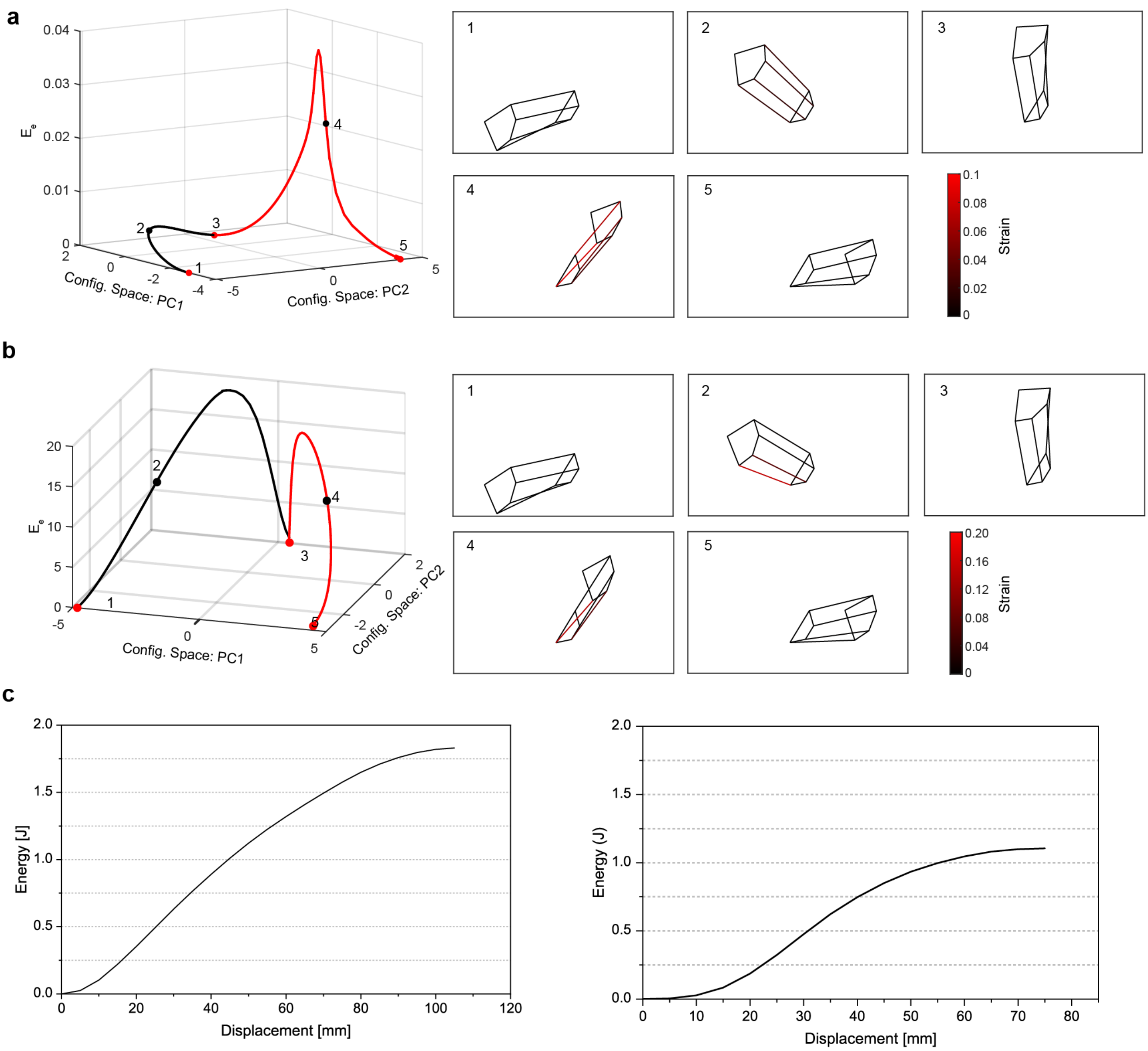

Supplementary Fig. 5. **The comparison of the MEP obtained by the truss model and beam model. a** The MEP obtained by the truss model. The strain of the Config. 4 is much larger, so the strain of the others is not obvious. **b** The MEP obtained by the beam model. **c** The energy curve obtained by integrating the experimental force curve in Fig. 5. The energy barrier in the physical experiment between the stable state 1 and 2 is higher, which matched better with the beam model since the elastic bars made of TPU of the prototype deform as beam elements.

Algorithm 1 Nudged Elastic Band

Given the initial state $\mathbf{C}_1$ and final state $\mathbf{C}_N$, tolerance η, step stp;
Initialize $\mathbf{C}_k^0, k = 2, ..., (N-1)$ by linear interpolation between $\mathbf{C}_1$ and $\mathbf{C}_N$
while $\sigma_k^{s,\|}$, $\sigma_k^{e,\perp} < \eta$ **do**
  for $k = 2, ..., N-1$ **do**
    if $E_e(\mathbf{C}_{k+1}) \geq E_e(\mathbf{C}_k) \geq E_e(\mathbf{C}_{k-1})$ **then**
      $u_k = \mathbf{C}_{k+1} - \mathbf{C}_k$
    else if $E_e(\mathbf{C}_{k+1}) \leq E_e(\mathbf{C}_k) \leq E_e(\mathbf{C}_{k-1})$ **then**
      $u_k = \mathbf{C}_k - \mathbf{C}_{k-1}$
    else
      $\triangle E_{e_k}^{max} = max(|E_e(\mathbf{C}_{k+1}) - E_e(\mathbf{C}_k)|, |E_e(\mathbf{C}_k) - E_e(\mathbf{C}_{k-1})|)$
      $\triangle E_{e_k}^{min} = min(|E_e(\mathbf{C}_{k+1}) - E_e(\mathbf{C}_k)|, |E_e(\mathbf{C}_k) - E_e(\mathbf{C}_{k-1})|)$
      if $E_e(\mathbf{C}_{k+1}) > E_e(\mathbf{C}_{k-1})$ **then**
        $u_k = (\mathbf{C}_{k+1} - \mathbf{C}_k) \triangle E_{e_k}^{max} + (\mathbf{C}_k - \mathbf{C}_{k-1}) \triangle E_{e_k}^{min}$
      else
        $u_k = (\mathbf{C}_{k+1} - \mathbf{C}_k) \triangle E_{e_k}^{min} + (\mathbf{C}_k - \mathbf{C}_{k-1}) \triangle E_{e_k}^{max}$
      end if
    end if
    $\tau_k = \mathbf{u}_k / |\mathbf{u}_k|$
    $\sigma_k^{s,\|} = \tau_k' k_s(|\mathbf{C}_{k+1} - \mathbf{C}_k| - |\mathbf{C}_k - \mathbf{C}_{k-1}|)\tau_k$
    $\sigma_k^{e,\perp} = \nabla E_e(\mathbf{C}_k) - \tau_k' \nabla E_e(\mathbf{C}_k)\tau_k$
  end for
  $C^{k+1} = C^k + stp(\sigma_k^{s,\|} + \sigma_k^{e,\perp})$
end while

4.4 Parametric study about the influence of the stiffness of components on MEP

To explore whether the stiffness of the bars affects the multi-stability of the structure, a parametric study about the influence of the stiffness of components on MEP is performed on the 2P4B structure. We set two values of stiffness, 1 and 10, representing elastic materials like TPU and stiff materials like PLA. By default, only replace part of four bars with TPU material to provide deformations. The result is presented in Supplementary Fig. 6, where 0 and 1 in the legend denote the bar is stiff (the stiffness is set to 10) or elastic (the stiffness is set to 1), and four numbers indicate the stiffness of the corresponding four bars. We can observe that the stiffness of the bars only affects the energy barrier rather than the multi-stability of the structure. The more elastic bars, the lower the energy barrier. When the number of elastic bars is identical, different positions of the elastic bars would also affect the energy barrier, as the comparison of the curves in warmer colors and colder colors.

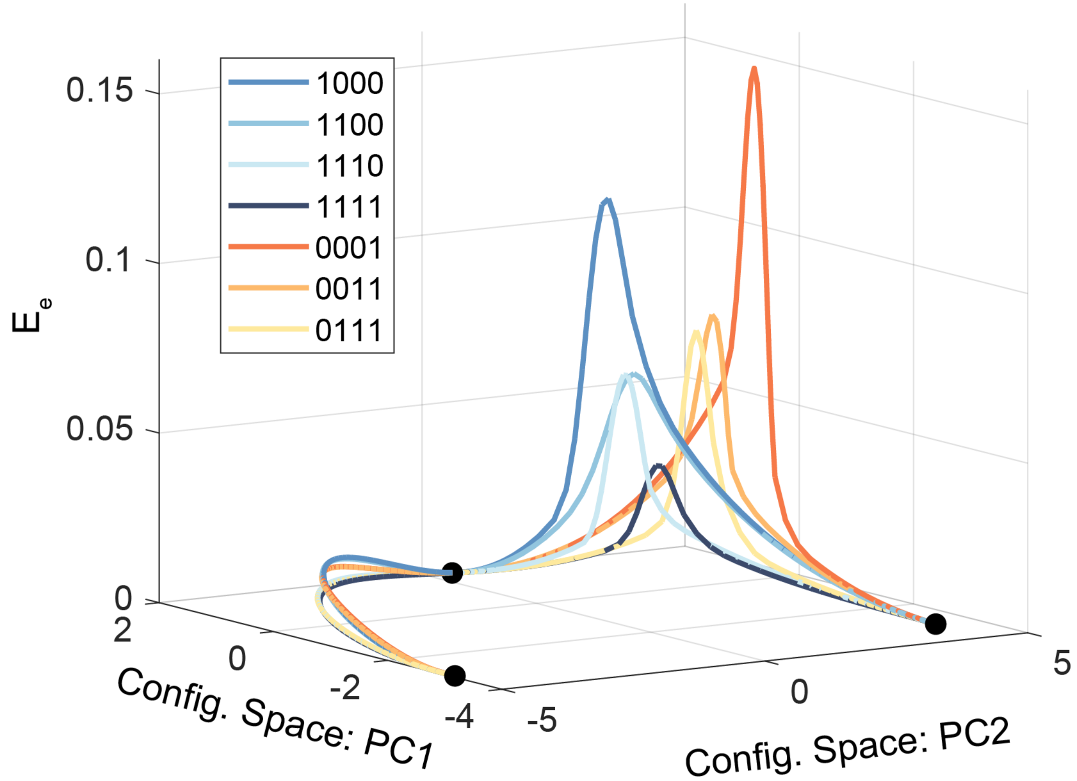

Supplementary Fig. 6. **A parametric study about the influence of the stiffness of components on the minimum energy path for the 2P4B structure.** In the legend, 0 and 1 denote the bar is stiff or elastic, and four numbers indicate the stiffness of the corresponding four bars.

5 Loop algorithm with the warm-start optimization

The multi-compatible structure can be designed with many other constraints or targets, such as tailoring the energy barrier or together with the actuation design for a more suitable actuator. However, achieving these further targets requires information on the MEP calculated by the NEB method, an iteration procedure. Therefore, we constructed a loop algorithm with the warm-start optimization, where the results obtained from a previous optimization are used as the initial guess or starting point for a subsequent optimization process. Generate a preliminary multi-stable structure by the first optimization *Opt*.1 and employ the NEB method to obtain the MEP. Then input this preliminary designed structure as the initial guess to the second optimization *Opt*.2, whose constraints based on the calculated MEP are added to the multi-compatibility constraints. The loop algorithms for the entire easy-to-actuate multi-stable structure design and energy barrier tailoring are shown in Supplementary Fig. 7. [6]

5.1 Algorithm for combining the structure design and actuation design

In Supplementary Fig. 7a: generate a multi-stable structure $G1$ that can achieve prescribed configurations in *Opt*.1 and calculate its reconfiguration path $MEP1$ through the NEB method. Input the structure design $G1$ as the initial guess of another optimization *Opt*.2, which satisfies the multi-compatibility constraints and the actuation design constraints based on $MEP1$. Then perform a kinematic simulation on the results of *Opt*.2, check whether the calculated actuator can actuate the designed multi-stable structure.

Supplementary Fig. 8 demonstrates design results $G2, AC1$ of *Opt*.2 withstanding the verification of the kinematic simulation. The red, green and blue areas denote three prescribed configurations of the moving plate. The red dashed lines denote the designed actuator, whose length decreases monotonically. The black dashed lines mean the endpoint of the calculated actuator is rigidly connected to the moving plate. Furthermore, the same optimization *Opt*.3 as *Opt*.2 can be performed more times on $G2, AC1$ until a more high-standard condition for the actuation converges, such as the ratio of the distance between the actuator mounting position on the moving plate and the reference point to the maximum geometric size of the moving plate.

5.2 Algorithm for tailoring the energy barrier

In Supplementary Fig. 7b: generate a multi-stable structure $G1$ that can achieve prescribed configurations and include the local stiffness in the constraints to guide the energy barriers to satisfy the energy tailoring target in *Opt*.1 (see Supplementary Information, Section 3 for the discussion about the relationship between the local stiffness and the energy barrier in detail). Calculate its reconfiguration path $MEP1$ through the NEB method. Check whether the results of *Opt*.1 with local stiffness design satisfy the energy tailoring condition. If it fails, input the structure and stiffness design $G1, K1$ as the initial guess of another optimization *Opt*.2, which satisfies the multi-compatibility constraints and the energy tailoring constraints based on $MEP1$. Then check whether the designs $G2, K2, MEP2$ from *Opt*.2 meet the energy condition. If it works, a multi-stable structure with prescribed energy barriers is obtained; if it does not work, take the MEP of the *Opt*. 2 result as the input parameters to carry out the second optimization again, until the output MEP satisfies the energy target.

Supplementary Fig. 9 demonstrates the results of *Opt*.1 and *Opt*.2 respectively. The target of energy tailoring is that the energy barrier of the reconfiguration from Config. 2 to 3 E_{23} is lower than that from Config. 1 to 2 E_{12}. Supplementary Fig. 9a presents that the results of the first optimization *Opt*. 1 did not satisfy the energy barrier tailoring target. Supplementary Fig. 9b demonstrates the final design results of the *Opt*. 2 satisfied the target. Furthermore, the same optimization *Opt*.3 as *Opt*.2 can be

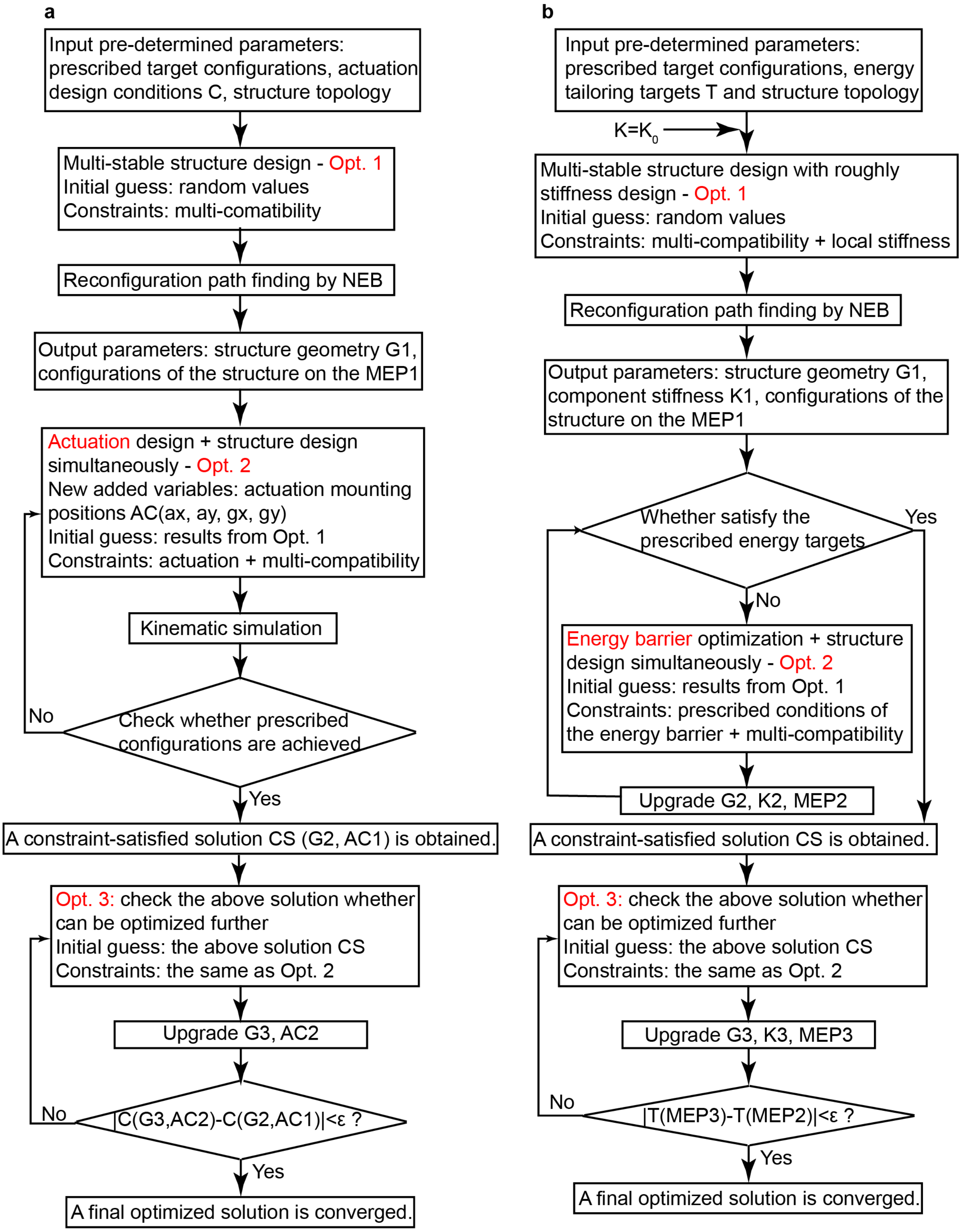

Supplementary Fig. 7. **The flow chart of the warm-start optimization, where the results obtained from a previous optimization are used as the initial guess or starting point for a subsequent optimization process. a** The warm-start optimization for the actuation design. **b** The warm-start optimization for tailoring energy barriers.

performed more times on the successful results $G2, K2, MEP2$ until a more high-standard condition for the energy barriers converges, such as the difference between E_{23} and E_{12} no longer decreases.

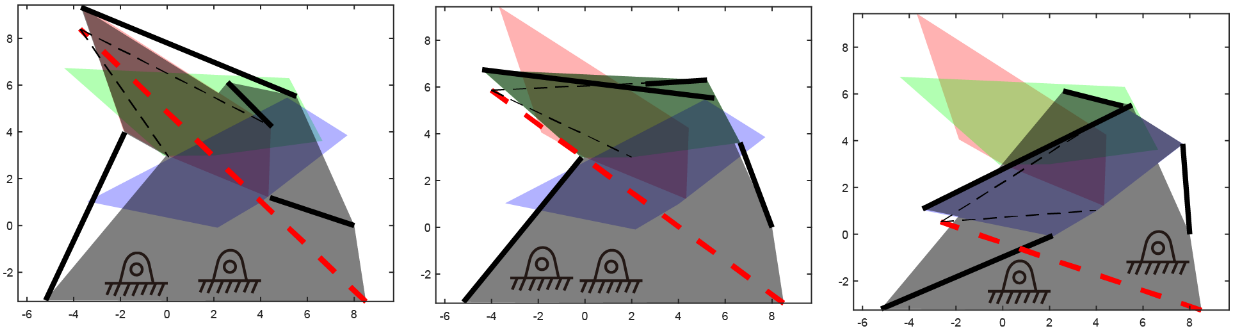

Supplementary Fig. 8. **The result of the double-optimization for the entire design procedure, i.e. combining the structure design and actuation design into one-step constrained optimization.** The red dashed lines denote the designed actuator, whose length decreases monotonically. The black dashed lines mean the endpoint of the calculated actuator is rigidly connected to the moving plate.

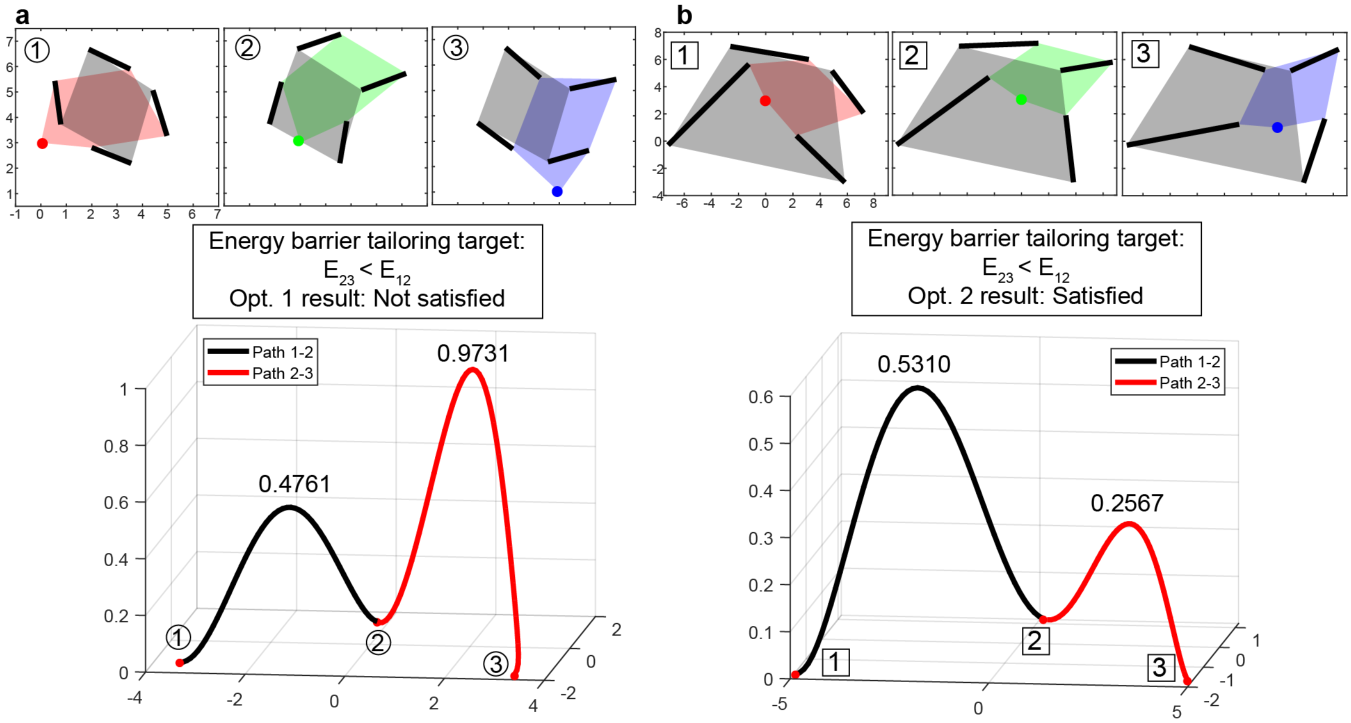

Supplementary Fig. 9. **The result of the double-optimization for tailoring the energy barrier.** The target of energy tailoring is that the energy barrier of the reconfiguration from Config. 2 to 3 is lower than that from Config. 1 to 2. **a** The result from Opt. 1. Although the local stiffness is considered in the constrained equations, the result did not satisfy the prescribed target of the energy barrier. **b** According to the MEP obtained from the design from Opt. 1, both the geometry of the structure and the stiffness of components are optimized to satisfy the target of energy tailoring.

6 Collision avoidance considerations

In order to make a designed planar multi-stable structure properly work without interference, one method is to assign the bars to different layers to avoid collision in the reconfigurations of the structure, the other method is to take the interference into account in the design stage to generate a real-2D structure. Based on the reference [7], we attempted to propose interference-free constraints for simple 2P4B structures and summarized the layering assignment algorithm for complex structures or structures with crank bars.

6.1 Interference-free constraints for simple 2P4B structures

Interference-free constraints ensure any two bars located on the same layer should be interference-free during kinematical motion. If there is a crank in the structure, i.e. one of the bars rotates 360 degrees, it would be inevitable to layer. Therefore, the limitations of the components' motions are defined firstly:

$$\max ang(\mathbf{B}_i^b\mathbf{G}_i^b, \mathbf{B}_j^b\mathbf{G}_j^b) < \pi, \quad i, j = 1, ..., nc, i \neq j, \quad b = 1, ..., nb \tag{44}$$

where nc and nb are the total numbers of configurations and bars, i and j denote the serial number of the configuration, b denotes the serial number of the bar.

Allowing for designing the profiles of links rather than using straight links connecting joints directly, multi-stable structures achieving the prescribed configurations would be designed in a much larger design space. Inspired by the reference [7], the lengths of bars have constraints as shown in Supplementary Fig. 10:

$$\left|\mathbf{B}_1^b\mathbf{G}_1^b\right| < \min\left\{\left|\mathbf{G}_1^b\mathbf{G}_1^a\right|, \left|\mathbf{G}_1^b\mathbf{G}_1^c\right|\right\} \tag{45}$$

where a and c denote the adjacent joints to the bth joint. In Supplementary Fig. 10, red shaded areas are the swept areas of the red bars, the grey segments in between the grounded pivot points denote the available space for reshaping the profiles of the black links.

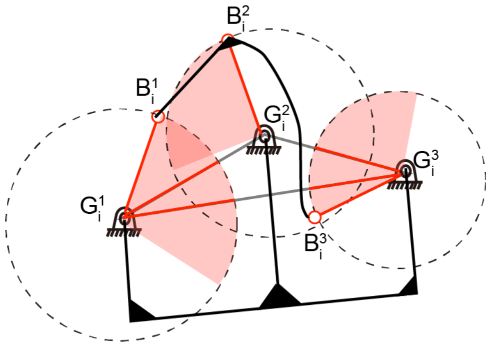

Supplementary Fig. 10. **The illustration of the interference-free constraints.** The red areas denote the swept areas of red bars, the grey segments in between the grounded pivot points denote the available space for reshaping the profiles of the black links.

6.2 Layering assignment

Assuming all of the bars are located on the same layer, the interference of components during the transformation is analyzed through the path calculated through the NEB method. Specifically, set one component as the reference system and plot the motion of the other components with respect to this

component until all components have been analyzed, which seems we stand on this component and observe the motions of the other components. Then in accordance with the above collision analysis, the components could be assigned to different layers following the below rules:

Rule 1: two adjacent links that are paired by the same joint should be assigned to different layers. [7]

Rule 2: two bars that collide with each other should be assigned to different layers.

Rule 3: if ith bar collides with kth joint, the ith bar should be assigned to the outermost layer (top or bottom) of all the bars connected by the kth joint. [8]

6.3 Layering example of the 3P6B structure

Here we take the three-plate-six-bar example in this paper as an example to illustrate. The three-plate-six-bar example has two kinds of components: plates and bars. According to the **Rule 1**, the plate and bar would not be at the same layer. Therefore, only the interference between plates and interference between bars would be considered. Supplementary Fig. 11 and Supplementary Fig. 12 are the interference analysis of the transformation from Config. 1 to Config. 2 and from Config. 2 to Config. 3.

The interference between plates is presented in Supplementary Fig. 11A. The black polyline represents the observed plate, and the colorful trajectories correspond to the bars in the same color as Supplementary Fig. 11B, where the polyline connecting all nodes on the same plate represents this plate because the coordinates of all nodes are designed exactly to satisfy multi-compatibility while the shape of plates can be modified to avoid interference.

Supplementary Fig. 11C shows the topology with bars in different colors, which are the legends of Supplementary Fig. 11D that shows the interference of bars. The observed bar is regarded as the reference system and one endpoint of the bar (denoted by *left*) is fixed as the reference system origin, so the reference bar only extends or shortens without rotation as the thicker part of the horizontal line segment. However, the thicker part could not demonstrate the extension of bars in the time dimension, which means whether the extension part of the observed bar collides with the other bars at the same time could not be determined by the overlap of the trajectories of other bars and the thicker part. In order to judge whether the collision happened, fix another endpoint of the bar (denoted by *right*) as the reference system origin, and observe the interference of bars again. Similarly, the same analysis could be conducted on the transformation from Config. 2 to Config. 3, as shown in Supplementary Fig. 12.

Therefore, we can extract the interference information from Supplementary Fig. 11 and 12, and summarize the interference as maps in Supplementary Fig. 13. According to the **Rule 2** and **Rule 3**, the structure has seven layers, and the assignment of components is as follows: plate 1 spans the first layer and the seventh layer. Plate 2 and plate 3 are at the third and fifth layers respectively. The second layer has bar 1 and bar 2. The fourth layer has bar 3 and bar 4. The sixth layer has bar 5 and bar 6.

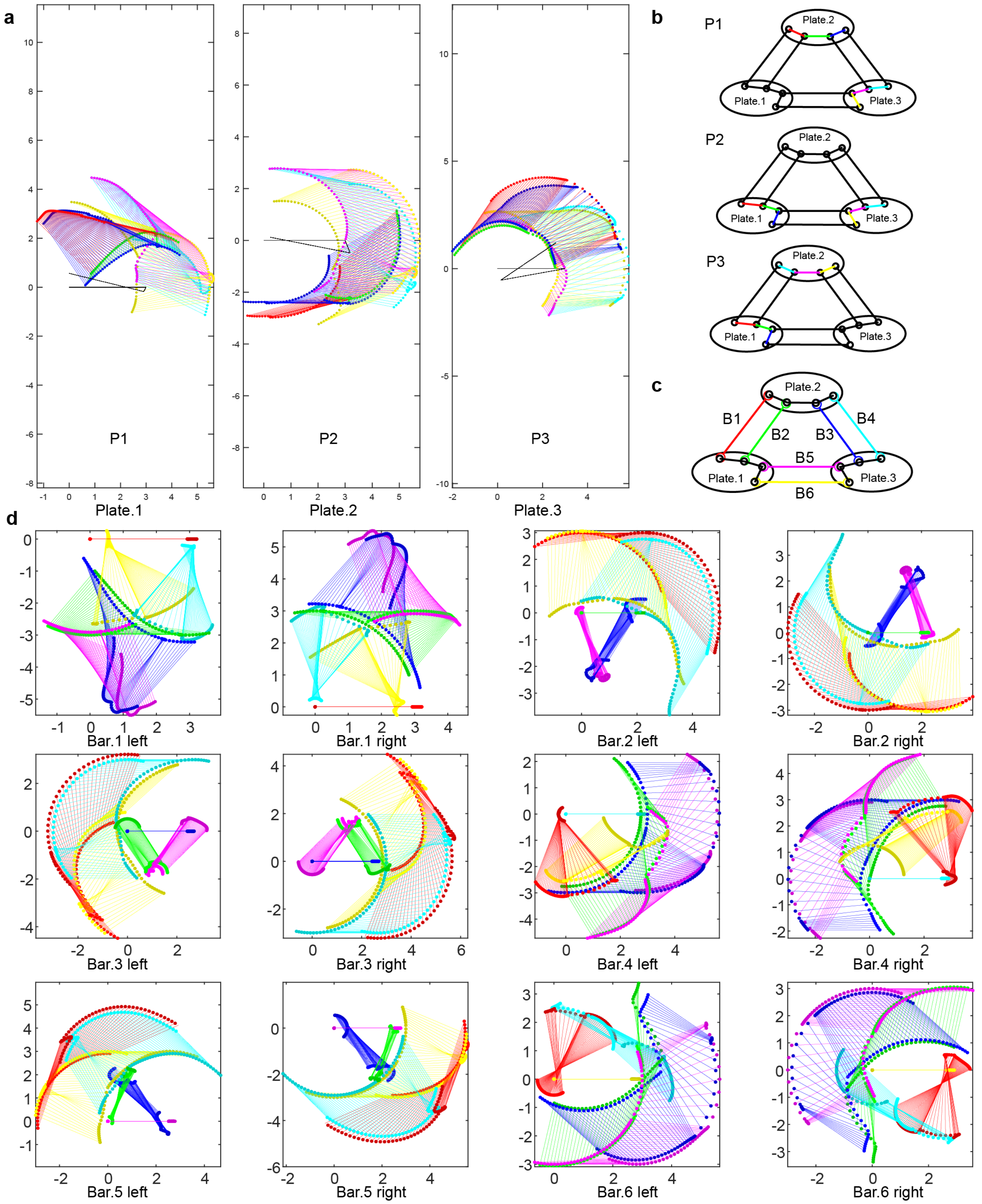

Supplementary Fig. 11. **The interference analysis during the transformation from Config. 1 to Config. 2 for the three-plate-six-bar structure. a** The interference between plates. The colorful lines denote trajectories of the bars in the same color as b. **b** The legends of a. **c** The topology of the structure and the legend of d. **d** The interference between bars. The colorful lines denote trajectories of the bars in the same color as c.

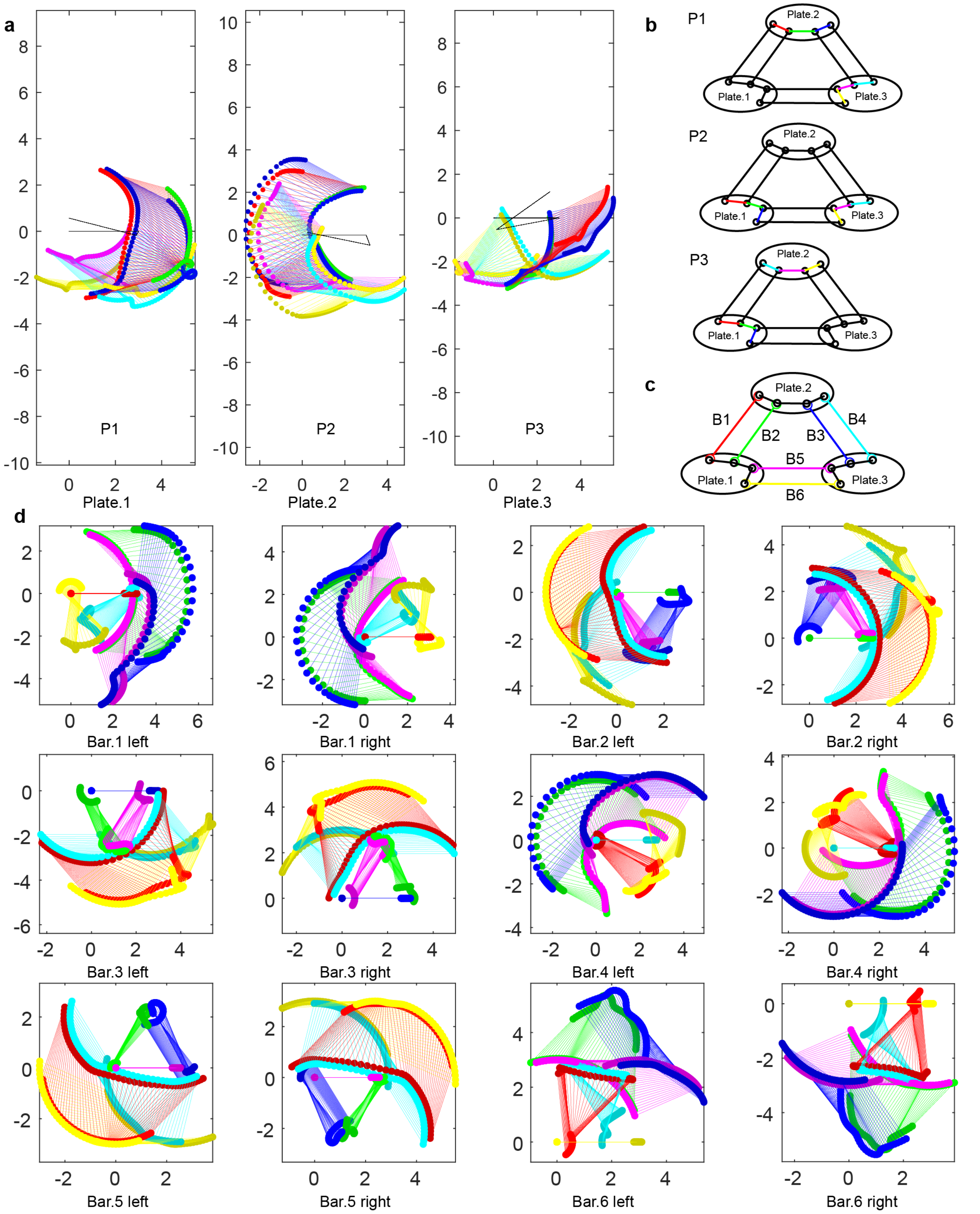

Supplementary Fig. 12. **The interference analysis during the transformation from Config. 2 to Config. 3 for the three-plate-six-bar structure. a** The interference between plates. The colorful lines denote trajectories of the bars in the same color as b. **b** The legends of a. **c** The topology of the structure and the legend of d. **d** The interference between bars. The colorful lines denote trajectories of the bars in the same color as c.

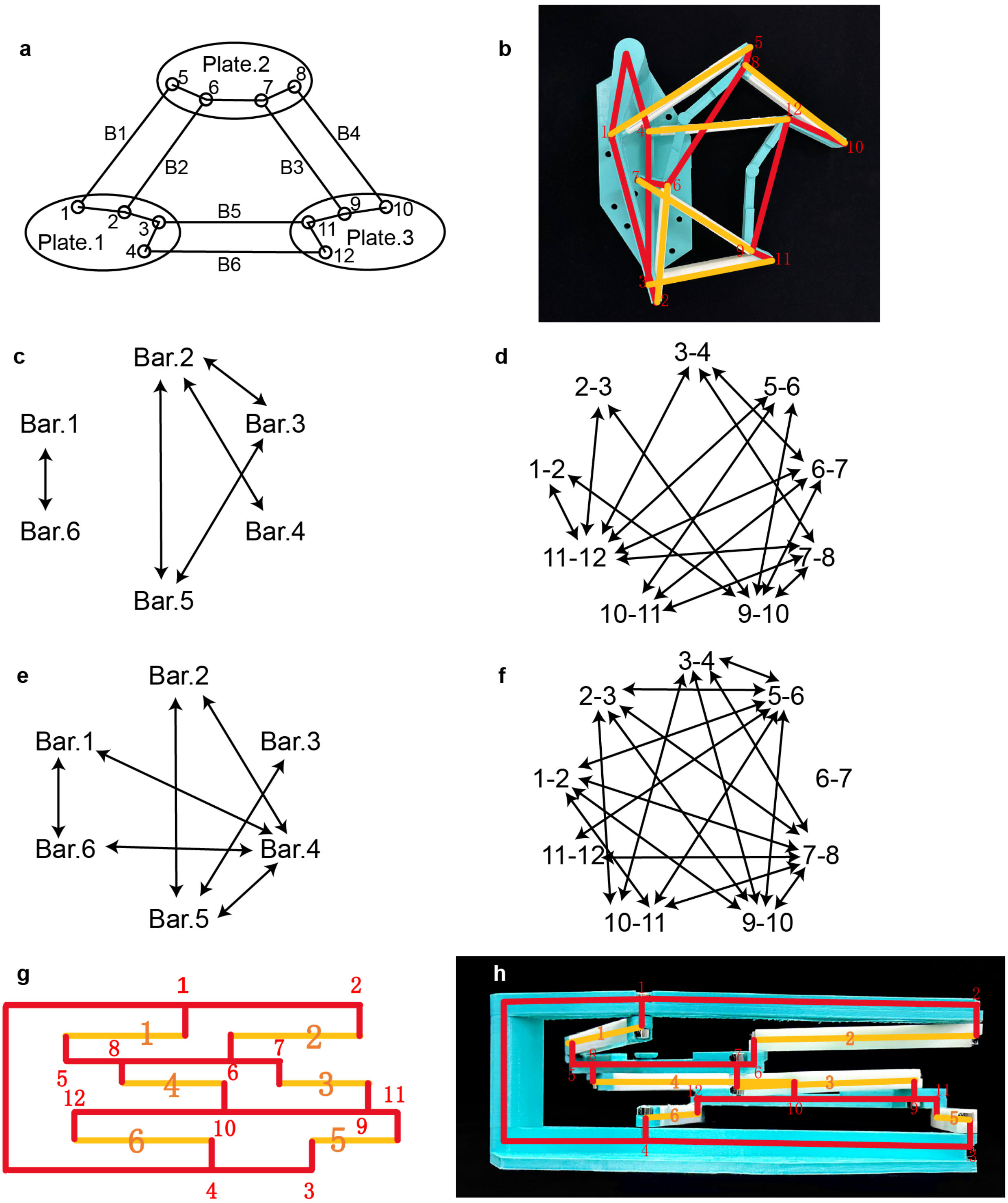

Supplementary Fig. 13. **The analysis of the interference. a** Topology of the 3P6B structure and the label of components. The label beginning with the letter B denotes the mark numbers of the bars. The numbers on the plates are utilized to represent the plates. **b** The physical model with the node mark. The red line represents the plate, while the orange ones denote the bars. **c** The analysis of interference between bars from Config. 1 to Config. 2. **d** The analysis of interference between plates from Config. 1 to Config. 2. **e** The analysis of interference between bars Config. 2 to Config. 3. **f** The analysis of interference between plates from Config. 2 to Config. 3. **g** The results of layering from c-f. **h** The physical model corresponds to g.

7 Simulation in MATLAB

The three-dimensional bar-and-body structure in this paper is modeled in terms of bodies and bars connected by universal joints. The universal joint has two axes (ax_1 and ax_2) which could form any angle. The bars with universal joints are modeled by tetrahedrons, and the bodies are also subdivided into tetrahedrons. Then the constituents of these tetrahedrons are pin-jointed bars, as shown in Supplementary Fig. 3B. All the bars mentioned below refer to pin-jointed bars.

The simulation uses an explicit time integration method, as in [9]. A general form of the equations of motions could be written as

$$\mathbf{M}\ddot{\mathbf{x}} + \mathbf{C_d}\dot{\mathbf{x}} + \mathbf{R}(\mathbf{x}) = \mathbf{P} \tag{46}$$

where $\mathbf{M}$ is a mass matrix, $\mathbf{C_d}$ is a damping matrix, $\mathbf{R}(\mathbf{x})$ denotes the vector of the internal forces and $\mathbf{P}$ contains the time-dependent prescribed loads.

The approximation of the time derivatives within a given time step could be expressed by the central difference quotient using the displacements at different times,

$$\dot{\mathbf{x}}_i = \frac{\mathbf{x}_{i+1} - \mathbf{x}_{i-1}}{2\Delta t} \tag{47}$$

$$\ddot{\mathbf{x}}_i = \frac{\mathbf{x}_{i+1} - 2\mathbf{x}_i + \mathbf{x}_{i-1}}{(\Delta t)^2} \tag{48}$$

Replacing $\dot{\mathbf{x}}_i$ and $\ddot{\mathbf{x}}_i$ in Eq. (46), $\mathbf{x}_{i+1}$ could be expressed by $\mathbf{x}_i$ and $\mathbf{x}_{i-1}$, which has been obtained in the past time steps,

$$\mathbf{M}(\mathbf{x}_{i+1} - 2\mathbf{x}_i + \mathbf{x}_{i-1}) + \frac{\Delta t}{2}\mathbf{C_d}(\mathbf{x}_{i+1} - \mathbf{x}_{i-1}) + (\Delta)^2\mathbf{R}(\mathbf{x_i}) = (\Delta)^2\mathbf{P}_i \tag{49}$$

$$(\mathbf{M} + \frac{\Delta t}{2}\mathbf{C_d})\mathbf{x}_{i+1} = (\Delta)^2(\mathbf{P}_i - \mathbf{R}(\mathbf{x_i})) + \frac{\Delta t}{2}\mathbf{C_d}\mathbf{x}_{i-1} + \mathbf{M}(2\mathbf{x}_i - \mathbf{x}_{i-1}) \tag{50}$$

where the mass matrix $\mathbf{M}$ and the damping matrix $\mathbf{C_d}$ are in diagonal form and constant.

Considering the initialization of Eq. (47)-(48), $\mathbf{x}_0$ means the initial configuration but the values of the displacements $\mathbf{x}_{-1}$ have to be determined in order to start the integration process in a consistent way. These displacements could be computed from the initial values $\ddot{\mathbf{x}}_0$ and $\dot{\mathbf{x}}_0$ based on a second-order accurate TAYLOR series expansion, the relation

$$\mathbf{x}_{-1} = \mathbf{x}_0 - \Delta t\dot{\mathbf{x}}_0 + \frac{(\Delta t)^2}{2}\ddot{\mathbf{x}}_0 \tag{51}$$

could be obtained where the velocity $\dot{\mathbf{x}}_0$ or the acceleration $\ddot{\mathbf{x}}_0$ could be set to zero vectors according to the physical simulation situation. Mostly, set the initial velocity as zero and the remaining $\ddot{\mathbf{x}}_0$ could be derived from Eq. (46).

To summarize, the simulation procedure starts by computing the initial values for $\ddot{\mathbf{x}}_0$ and $\dot{\mathbf{x}}_0$. Then each iteration computes the current deformation, applies the current external load, derives the current $\mathbf{x}$, $\ddot{\mathbf{x}}$ and $\dot{\mathbf{x}}$, and goes to the next iteration until it finishes the loop.

8 Fabrication

All 3D printing tasks use the Ultimaker S-series 3D printers. There are two methods of fabrication for the joints in the multi-stable structures. One way is to use out-of-shelf revolute joints to assemble 3D-printed components when the interference of components exists during the transformation, as shown in Supplementary Fig. 14a. There is a bushing in each hole of the component, and the shaft crosses

two components, forming a revolute joint. Two spacing rings are added to the endpoints of the shaft to prevent the shaft from slipping out, as shown in Supplementary Fig. 14b. Because the bushing and the shaft are out-of-shelf parts, there is little friction in the rotation motion between them. When there is no interference, the whole physical model can be integratedly 3D printed, as shown in Supplementary Fig. 14c. In Supplementary Fig. 14d, the parts made of carbon fiber with high stiffness are fully wrapped by TPU to avoid the split between them, while the revolute joint is made of thin layers of TPU [10].

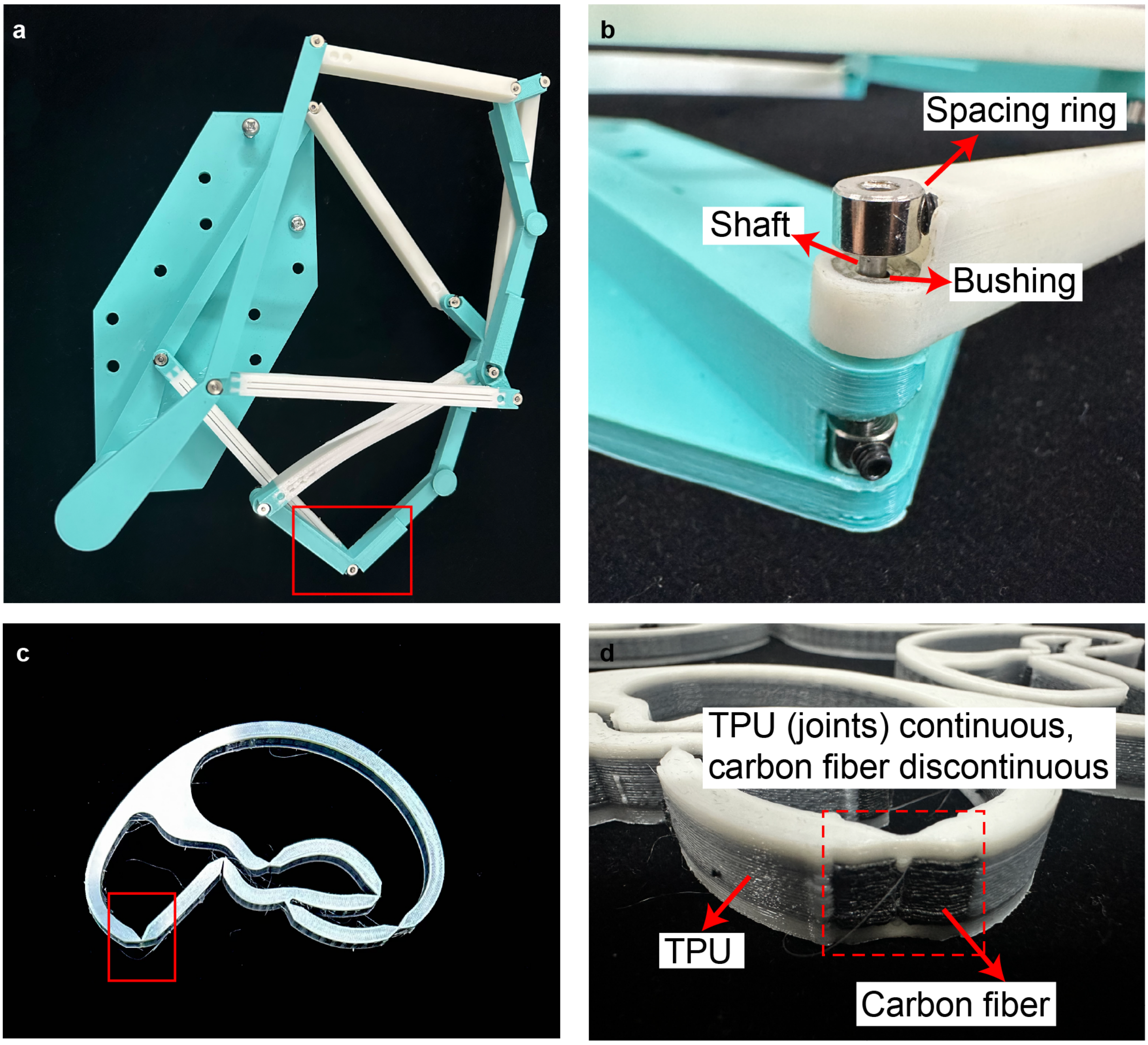

Supplementary Fig. 14. **Two methods of fabrication for the multi-stable structures.** One is 3D printing components and assembling them, as shown in a. The other is integratedly 3D printing the whole physical model, as shown in c. **a** When the interference of components exists, layering is necessary. The components are 3D printed with PLA or TPU and assembled through the revolute joint fabricated, as shown in a. **b** The disassembly demonstration of a revolute joint. There is one bushing in each hole of the components. The shaft crosses two bushings forming a frictionless revolute joint, and two spacing rings are added to two endpoints of the shaft to prevent the shaft from slipping. **c** For the real-2D case, there is no interference of components. The physical model can be integratedly printed. **d** The black layer is made of carbon fiber, and the white layer is made of TPU. The parts of carbon fiber are wrapper by TPU and the revolute joint is made of thin layers of TPU.

9 Experiment setup

In order to verify the actuation design and characterize the multi-stability behavior, a dedicated setup has been built as shown in Supplementary Fig. 15a. It comprises a force sensor that can apply a maximum force of 29.4N with an accuracy of $9.8*10^{-3}$N, a lead screw for controlling the displacement, and a platform to fix the multi-stable structure. The force sensor is fixed on the slider on the lead screw and connected to the actuator location of the structure by strings. We rotate the handwheel driving the slider away from the model, and a pull force is applied. The laptop records the force-displacement data. Supplementary Fig. 15b demonstrates an equally scaled-up gripper to measure the gripping force data by the same force sensor as a. The gripping force is measured when the distance between the end effectors varies.

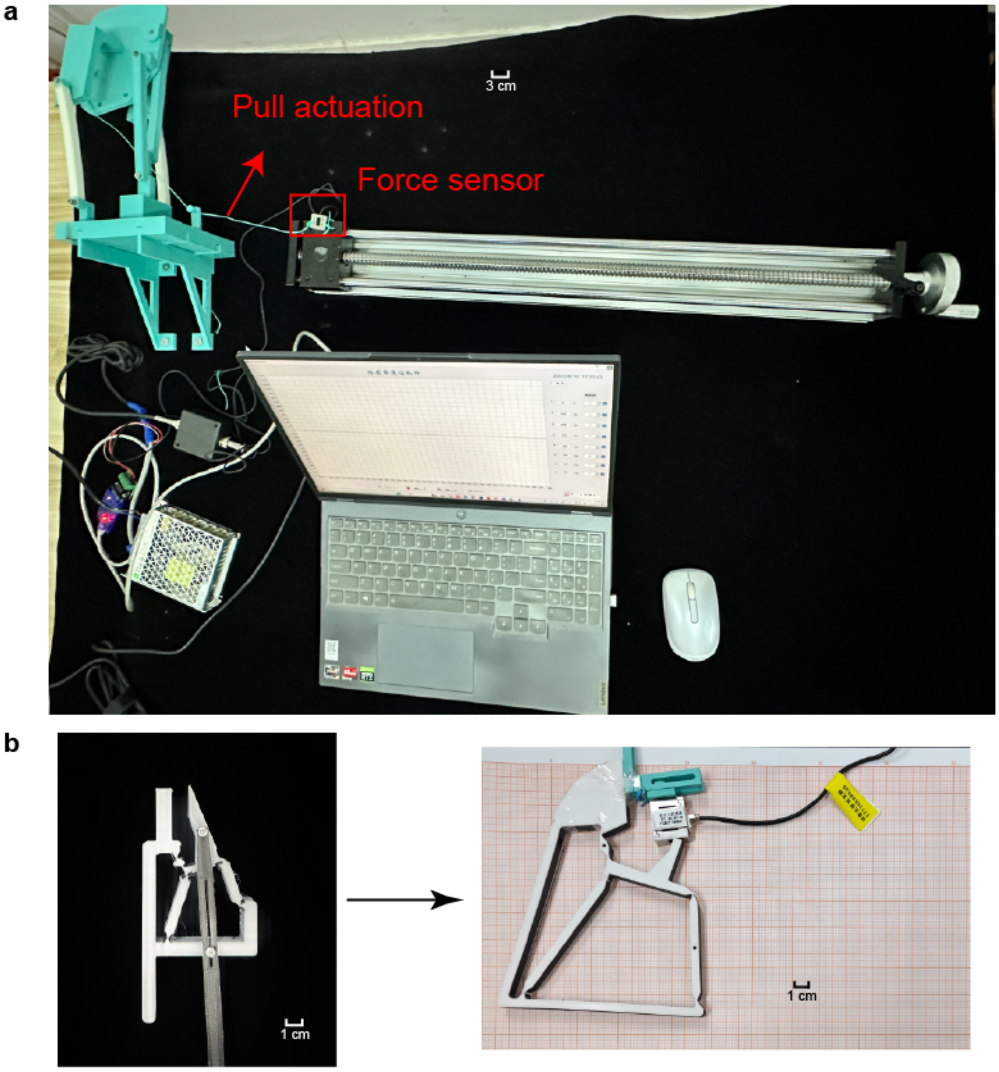

Supplementary Fig. 15. **The experiment setup. a** The actuation experiment of 2P4B and 3P6B structures. It is made up of a force sensor that can apply a maximum force of 29.4N with an accuracy of $9.8*10^{-3}$N, a lead screw for controlling the displacement, and a platform to fix the multi-stable structure. The force sensor is fixed on the slider on the lead screw and connected to the actuator location of the structure by strings. We rotate the handwheel driving the slider away from the model, and a pull force is applied. The laptop records the force-displacement data. **b** An equally scaled-up gripper is constructed to measure the gripping force data by the same force sensor as a. The gripping force is measured when the distance between the end effectors varies.

10 Extended studies on the multi-compatibility design method

10.1 Extension of the method on a 3D example

A tri-stable structure in 3D with topology in Supplementary Fig. 16a has been designed. As Section 2 introduces, the target configurations are prescribed by the coordinates of the reference points $\mathbf{P}$ and rotations θ, and use the rotation is expressed by the axis-angle representations, where the first three values form the rotation axis vector and the last value denotes the rotation angle around the rotation axis. The prescribed configurations are listed as follows:

$$\mathbf{P} = \begin{bmatrix} \mathbf{P}_1^1, \mathbf{P}_1^2, \mathbf{P}_1^3 \\ \mathbf{P}_2^1, \mathbf{P}_2^2, \mathbf{P}_2^3 \\ \mathbf{P}_3^1, \mathbf{P}_3^2, \mathbf{P}_3^3 \end{bmatrix} = \begin{bmatrix} [0,0,0],[0,15,10],[9,15,-5] \\ [0,0,0],[9,15,-5],[-9,15,-5] \\ [0,0,0],[-9,15,-5],[0,15,10] \end{bmatrix} \tag{52}$$

$$\theta = \begin{bmatrix} \theta_1^1, \theta_1^2, \theta_1^3 \\ \theta_2^1, \theta_2^2, \theta_2^3 \\ \theta_3^1, \theta_3^2, \theta_3^3 \end{bmatrix} = \begin{bmatrix} [0,1,0,0°],[0,1,0,0°],[0,1,0,0°] \\ [0,1,0,0°],[0,1,0,120°],[0,1,0,120°] \\ [0,1,0,0°],[0,1,0,120°],[0,1,0,120°] \end{bmatrix} \tag{53}$$

where the superscript denotes the number of components, and the subscript denotes the number of configurations.

Because the interference of all components is unavoidable in 3D, the physical model is hard to manufacture, and only simulations (as in Section 7) are conducted. In the simulation algorithm, all bars are modeled by the truss element. The structure is actuated by pulling every node from the initial configuration to the target configuration until across all prescribed configurations and its energy is recorded in Supplementary Fig. 16b.

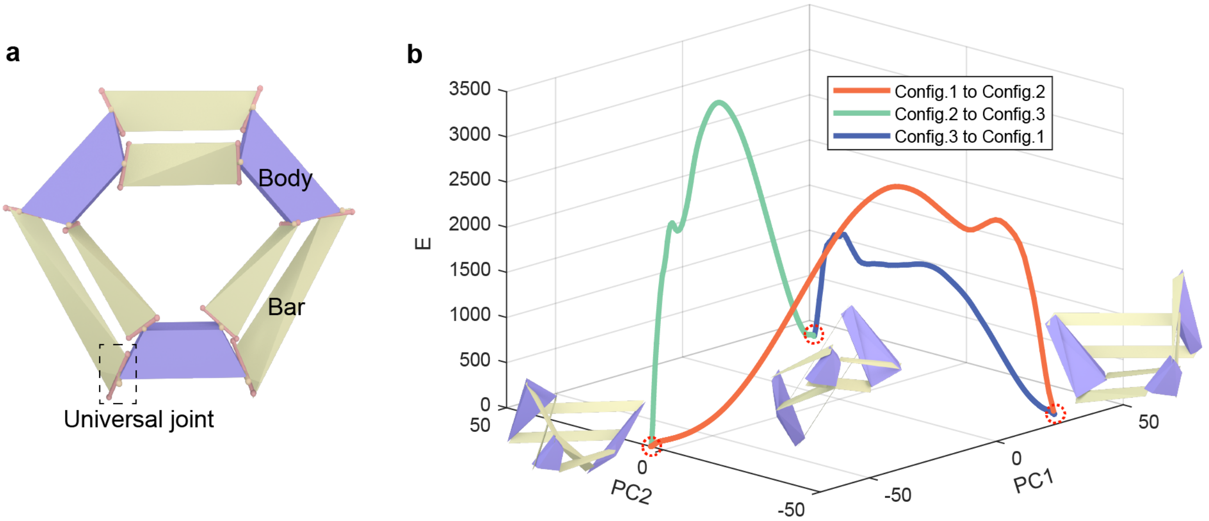

Supplementary Fig. 16. **3D tri-stable structure design and its simulation in MATLAB. a** Topology of a three-body-six-bar structure in 3D, the purple ones are bodies, the yellow ones are bars, and all joints are universal joints. **b** Energy curve of the tri-stable structure from the simulation. The tri-stable structure is actuated by pulling every node from the initial configuration to the target configuration until across all prescribed configurations and its energy curve is recorded.

10.2 Properties of the design method

Solving for a multi-stable structure is a nonlinearly constrained optimization problem, and the condition of solutions is hard to calculate analytically or predict. We could obtain one multi-compatible

structure design achieving prescribed configurations once the nonlinear constrained optimization problem converges to a solution. Then we conduct a large number of similar numerical experiments with different initial-guess inputs through MATLAB built-in function "fmincon" and use the statistical approach to evaluate the probability of converging to a solution that represents the difficulty level of a certain design problem.

10.2.1 Design space of the design method

How design parameters influence the multi-stable structure design is explored. Design parameters refer to the number of components, the percentage of reference points, and the degrees of static indeterminacy, and the design space of the multi-compatibility design means the maximum number of stable states that can be designed and the distribution of total stable state number. Although this design method could be applied in 2D and 3D, the interference of numerous components in 3D leads to the failure of a physical model. So we focus on the structure in 2D and the components will be constrained in a plane to be plates.

To explore the influence of the number of plates, one category of topology that all plates are connected by bars rather than by plates with zero DOF is adopted. In addition, all plates are reference plates, and bars can not be reference ones by default, which ensures the structure has the same percentage of the number of reference plates. The topologies of the structure that we studied are shown in Supplementary Fig. 17a, which have 2, 3, and 4 plates respectively. For these three structures, we prescribed 3, 4, 5, 6 target configurations to explore the maximum number of stable states that we can design. In addition, the study about the distribution of total stable state number was conducted by exerting a perturbation to a multi-stable structure that could achieve 3 prescribed configurations and explored the structure whether has additional stable states, including global and local stable states. The former study result is shown in Supplementary Fig. 17c while the latter one is shown in Supplementary Fig. 17f. The increasing number of plates represents the increasing complexity of structures, so it is more difficult to design a multi-stable structure that can achieve more target configurations. However, the structures with higher complexity have more possibility for different stable states, so the probability of distribution of total stable states is more dispersed and the peak of the probability distribution moves to larger values. We conducted the study on a structure consisting of four plates and nine bars, which DOF is zero, shown as the third one in Supplementary Fig. 17a. We changed the percentage of the number of reference plates, from 1/4 to 4/4. The minimum value 1/4 refers to only one plate fixed at the ground and other components free, while the maximum value 4/4 refers to all plates are prescribed to target configurations. Similarly, we expected they can achieve 3, 4, 5, 6 target configurations to explore the maximum number of stable states that we can design. The result is shown in Supplementary Fig. 17d. We can see that when the number of reference plates in one structure increases, the number of designed stable states decreases. More reference plates in the structure mean the description of the target configurations is more critical, so the higher requirement on the fitting target configurations would be at the expense of the number of prescribed stable states. Similarly, the experiment about the distribution of total stable states has been carried out, the result is shown in Supplementary Fig. 17g. We can see that changes in the percentage of the number of reference plates do not significantly affect the distribution of the number of total stable states.

The numerical analysis about the influence of DSI was conducted on the structure with just two plates. The DSI increases from 0 to 4 by adding bars between these two plates, as shown in Supplementary Fig. 17b. And these two plates are the reference plates during all the numerical experiments about DSI, which ensures the number of plates and the percentage of the number of reference plates remain unchanged while only DSI changes. The influence of DSI on the designed stable states is shown in Supplementary Fig. 17e and that on the total stable states is shown in Supplementary Fig. 17h. We can see that when DSI increases, which means the structure could be more resistant to impact, the

number of designed stable states is in decline. Another phenomenon is that higher DSI makes the peak of the probability distribution of total stable states move to a larger value.

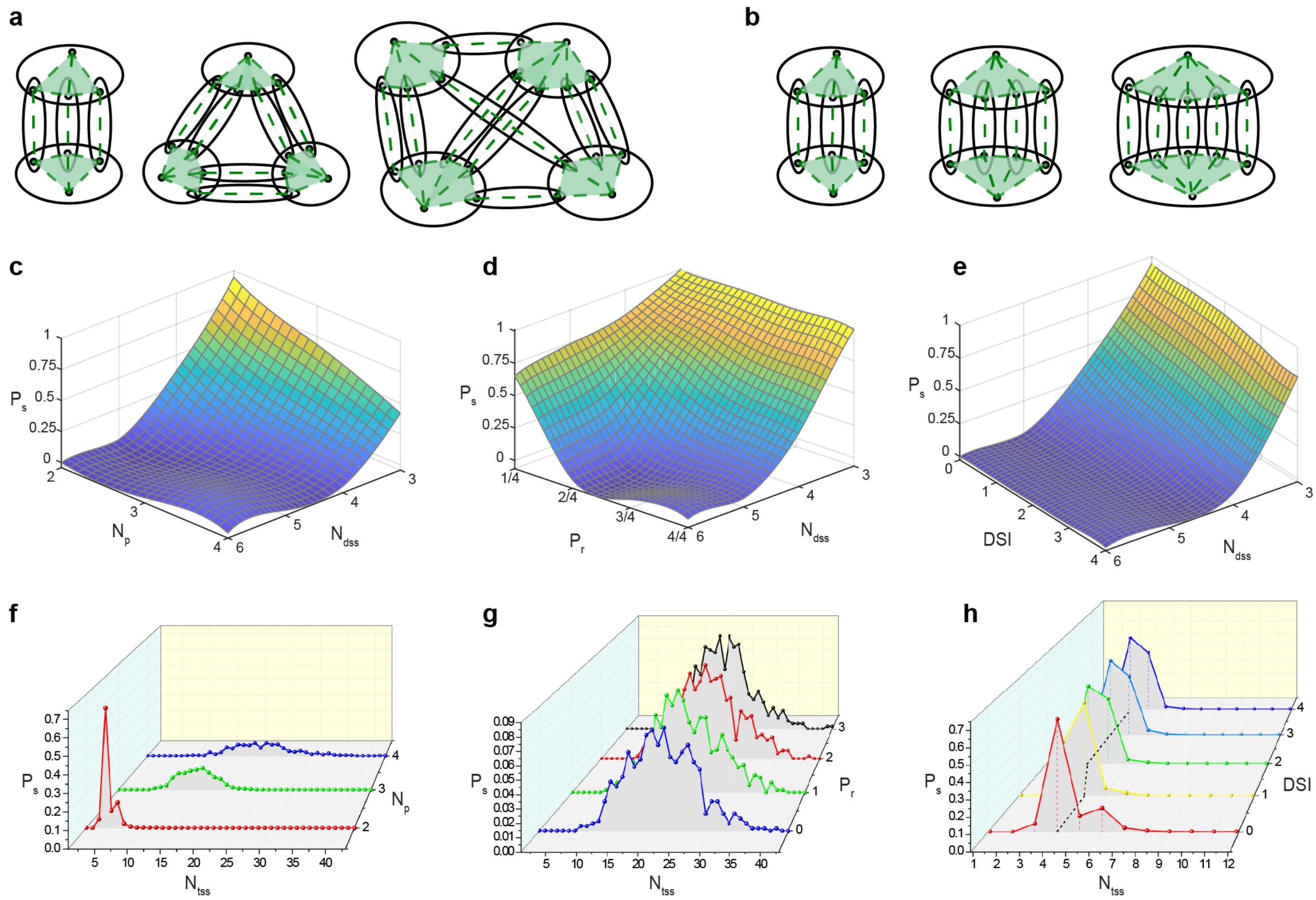

Supplementary Fig. 17. **Properties of the multi-compatibility design method. a** Schematic diagram of objects in parametric study about different number of plates and reference points. **b** Schematic diagram of objects in parametric study about different degrees of static indeterminacy (DSI). **c-e** Statistic results: the maximum number of designed stable states N_{dss} is influenced by the number of plates N_p, the percentage of the number of reference plates P_r, degrees of static indeterminacy DSI. **f-h** Statistic results: the number of total stable states N_{tss}, including global stable states and local stable states, which is also influenced by the number of plates N_p, the percentage of the number of reference plates P_r, degrees of static indeterminacy DSI.

10.2.2 The influence of the prescribed target

According to our observation, the prescribed target would influence the solutions of the inverse design, the local stiffness, and the number of additional stable states. Therefore, the parametric study about the solution to different prescribed targets is conducted.

Considering a bi-stable two-plate-three-bar structure is to be designed, two prescribed configurations are chosen from the following conditions: there is a dot matrix as Supplementary Fig. 18a and the topology is also plotted in fig. S9A, one plate is fixed at [0,0], the target of another plate is at the other positions in sequence, and the plate rotates 0, 45, and 90 degrees in sequence at each position.

There are 24 (3*8) prescribed target configurations in total and two target stable states would be two of the above configurations, then different combinations of the prescribed bi-stable configurations lead to different results, shown in Supplementary Fig. 18b-18d.

Then the numerical experiment of a more complex topology is also conducted. Considering a bi-stable three-plate-six-bar structure is to be designed, two prescribed configurations are chosen from the following conditions: there is a dot matrix as Supplementary Fig. 19a and the topology is also plotted in Supplementary Fig. 19a, one plate is fixed at [0,0], the target of the other two plates are at the other different positions in sequence, and each plate would rotate 0, 45, and 90 degrees in sequence at each position. There are 27 (3*3*3) prescribed target configurations in total and two target stable states would be two of the above configurations, then different combinations of the prescribed bi-stable configurations lead to different results, shown in Supplementary Fig. 19b-S19d.

As observed in the comparison between Supplementary Fig. 18 and Supplementary Fig. 19, the three-plate-six-bar structure could not converge to a solution in more conditions, because there are more constraints of multi-compatibility with the increasing complexity of the structure. The local stiffness of the three-plate-six-bar structure is generally lower than that of the two-plate-three-bar structure. The number of additional stable states is comparable between these two topologies. For a two-plate-three-bar structure, the number of additional stable states is obviously larger in some conditions as shown in red boxes in Supplementary Fig. 18d, caused by the symmetry of two prescribed configurations possibly.

10.3 Actuation design for a complex structure assembled by two quadra-stable unit cells

To explore the generality of the reconfiguration path finding and actuation design method, we studied whether a more complex structure assembled by quadra-stable unit cells is controllable during the reconfiguration. A structure assembled by two quadra-stable cells as shown in Supplementary Fig. 20a would have 16 stable states in total since each unit cell has 4 stable states and two of all stable states are prescribed as the target configurations for the assembly. The reconfiguration path between two target configurations is found by the NEB method as shown by the blue curve in Supplementary Fig. 20b, which predicted the structure preferred to experience 5 of all stable states to achieve the prescribed reconfiguration between two target configurations as shown in Supplementary Fig. 20c. Then the actuation design is conducted according to the found reconfiguration path. Supplementary Fig. 20d demonstrates the results of the simulation that actuates the assembled structure to reconfigure from Config. 1 to Config. 7 by the designed actuator denoted by the red dashed line, and the energy curve obtained by the simulation is presented by the purple curve in Supplementary Fig. 20b. We can observe that the reconfiguration process by the simulation (Supplementary Fig. 20)d matched well with the reconfiguration path calculated by the NEB method (Supplementary Fig. 20).

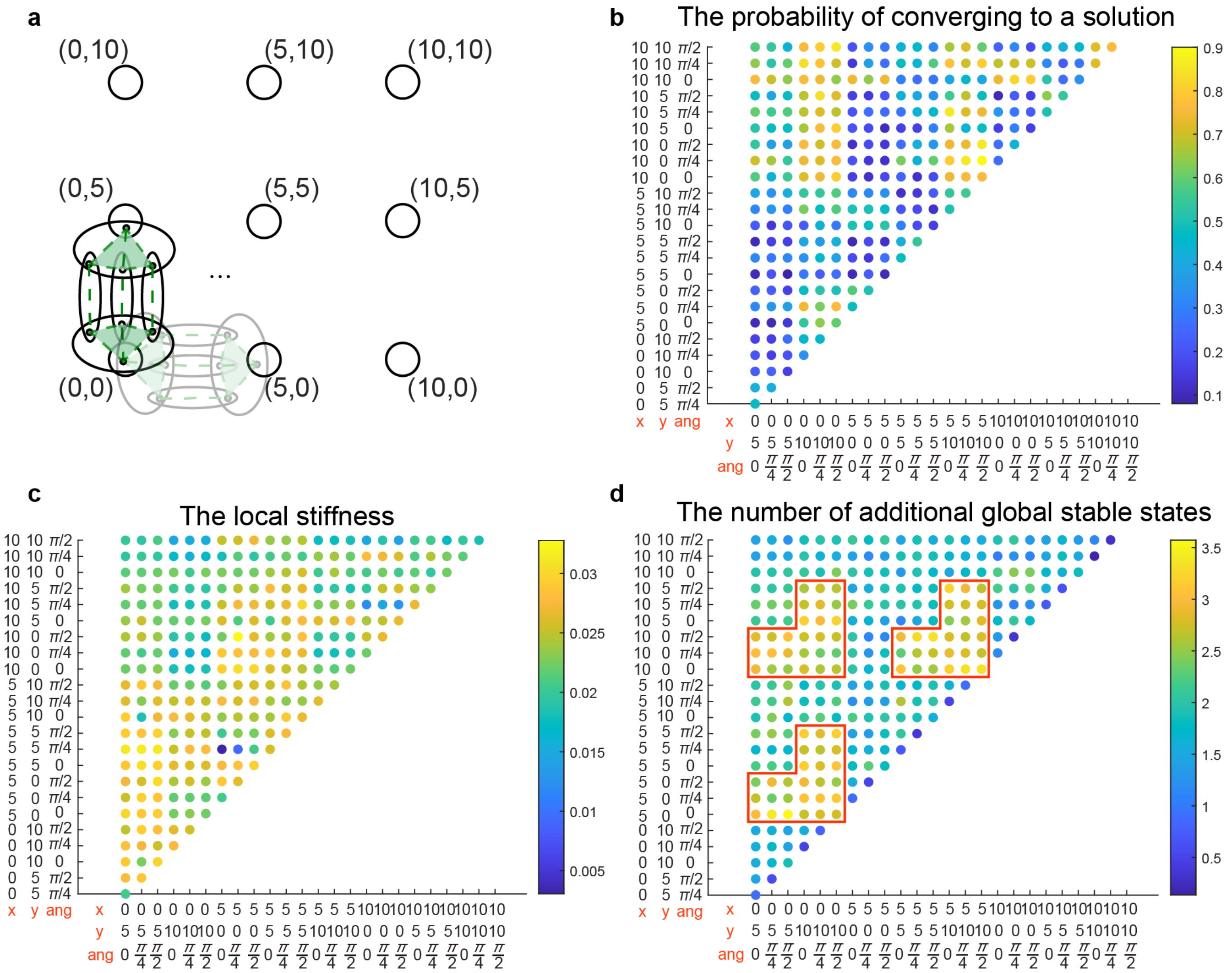

Supplementary Fig. 18. **The result of the parametric study about the influence of the prescribed target on designing the bi-stable two-plate-three-bar example. a** List 24 possible prescribed target configurations. One plate is fixed at (0,0), the other plate could be at the other 8 positions, and rotate 0 or 45 or 90 degrees, so there are 24 prescribed stable states chosen in total. **b** The influence of the prescribed targets on the probability of converging to a solution for the bi-stable two-plate-three-bar example. The x and y of each colorful dot represent two stable states, and the color means the probability of converging to a solution to design such a bi-stable structure. **c** The influence of the prescribed targets on the local stiffness for the bi-stable two-plate-three-bar example. The x and y have the same meaning as b, and the color means the average local stiffness of such a bi-stable structure. **d** The influence of the prescribed targets on the probability of converging to a solution for the bi-stable two-plate-three-bar example. The x and y have the same meaning as b, and the color means the number of additional global stable states when designing such a bi-stable structure.

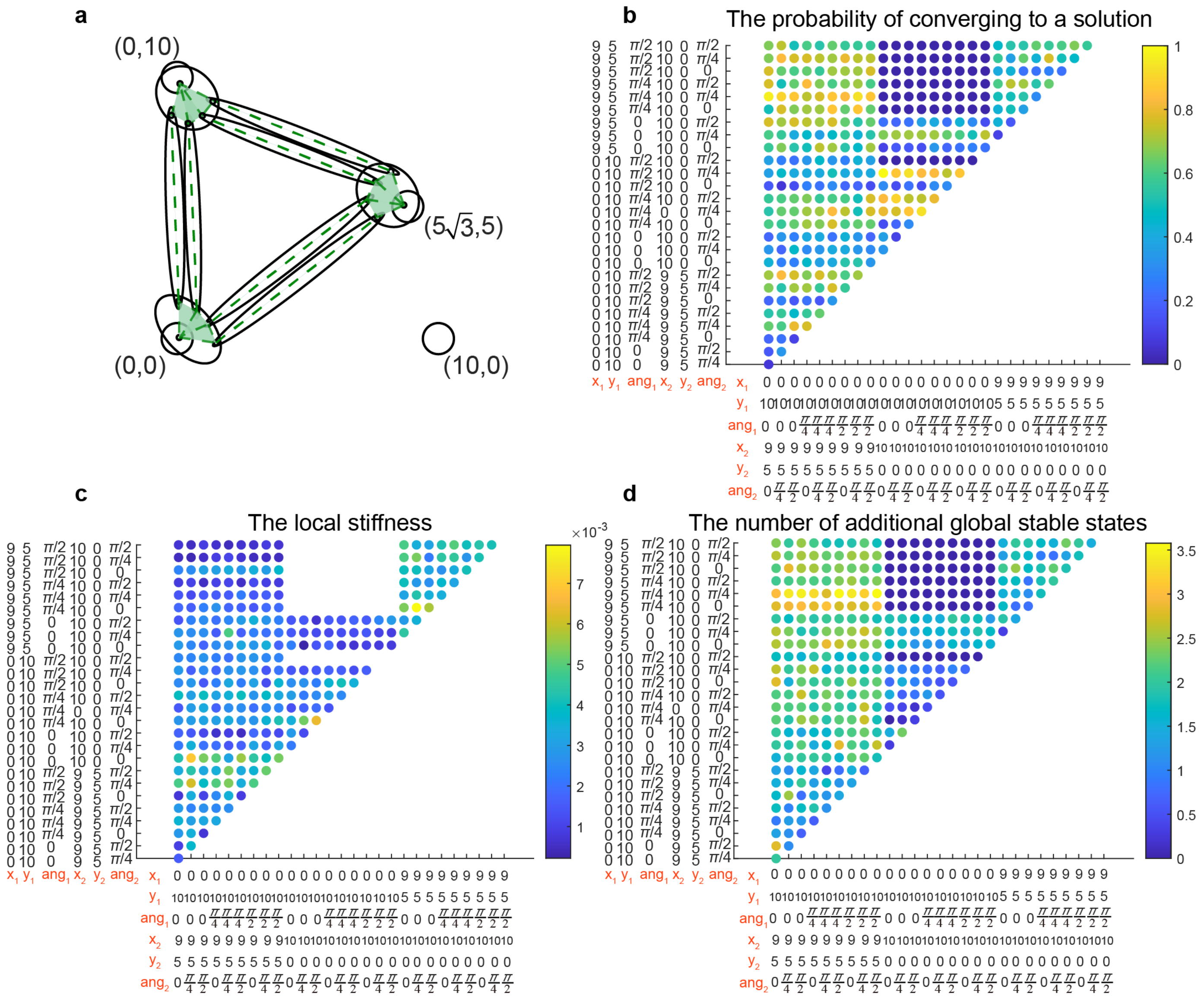

Supplementary Fig. 19. **The result of the parametric study about the influence of the prescribed target on designing the bi-stable three-plate-six-bar example. a** List 27 possible prescribed target configurations. One plate is fixed at (0,0), the other two plate could be at the other 3 positions, so there are 3 combinations, and each plate could rotate 0 or 45 or 90 degrees, so there are 27 (3*3*3) prescribed stable states chosen in total. **b** The influence of the prescribed targets on the probability of converging to a solution for the bi-stable three-plate-six-bar example. The x and y of each colorful dot represent two stable states, and the color means the probability of converging to a solution to design such a bi-stable structure. **c** The influence of the prescribed targets on the local stiffness for the bi-stable three-plate-six-bar example. The x and y have the same meaning as b, and the color means the average local stiffness of such a bi-stable structure. **d** The influence of the prescribed targets on the probability of converging to a solution for the bi-stable three-plate-six-bar example. The x and y have the same meaning as b, and the color means the number of additional global stable states when designing such a bi-stable structure.

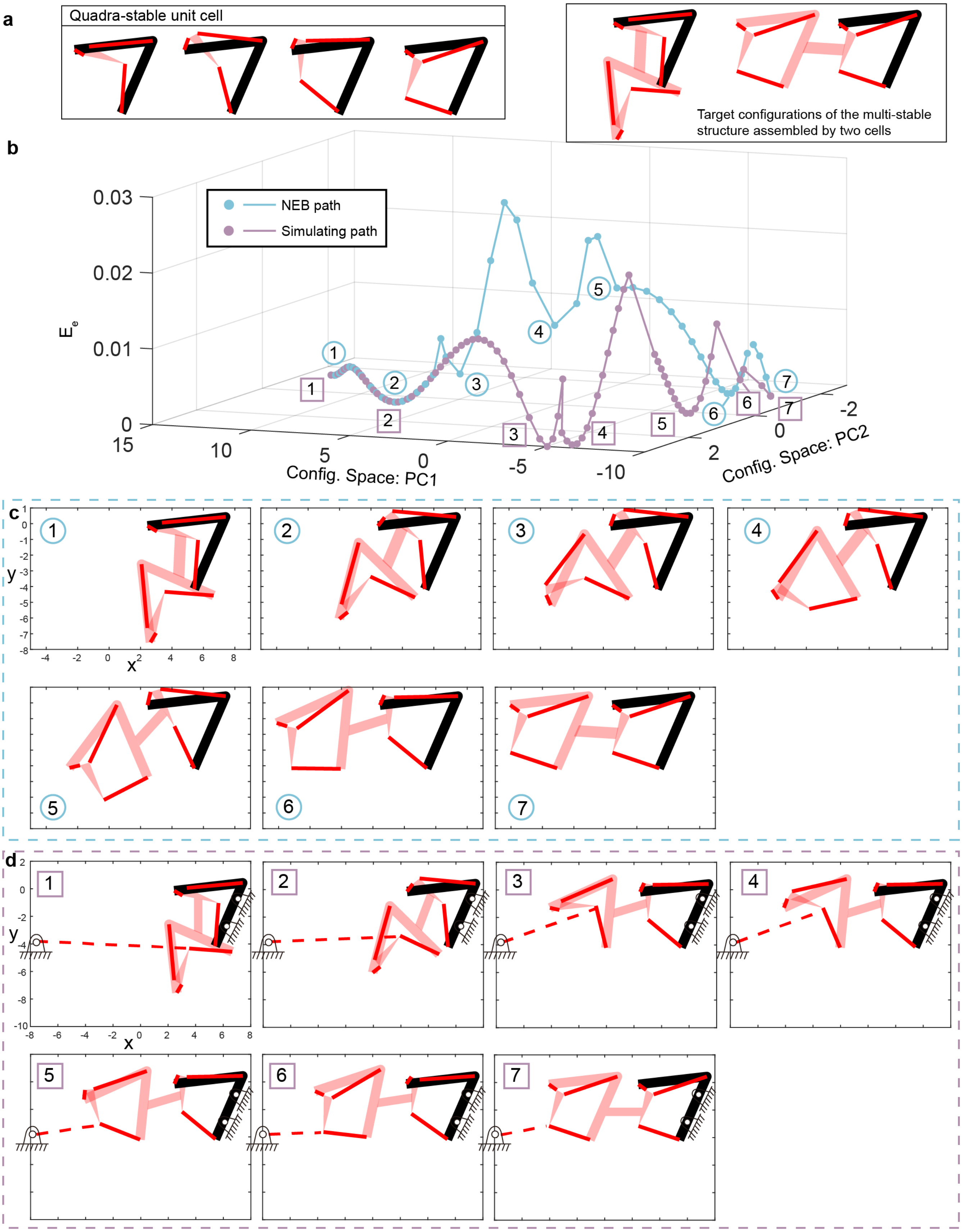

Supplementary Fig. 20 **The MEP of a two-unit-cell assembly, in which each unit cell has four stable states, is founded by the NEB method. a** Utilize two quadra-stable unit cells to assemble a multi-stable structure, the assembly would have 16 stable states in total for each unit cell has 4 stable states. Two of all stable states are prescribed as the target configurations. **b** The blue curve denotes the MEP founded by the NEB method, which predicted that the structure preferred to experience 5 of all stable states to achieve the prescribed reconfiguration from Config. 1 to 7. Then we conducted the actuation design, the actuator is shown as the red dashed line in c. The purple curve is the reconfiguration path through the simulation of the designed actuator. **c, d** The configurations on the reconfiguration path through the simulation matched well with those on the NEB path.

11 Supplementary information of the examples demonstrated with physical models

11.1 Prescribed target configurations of the physical models

The prescribed configurations for the two-plate-four-bar (2P4B) structure are listed as follows:

$$\mathbf{P} = \begin{bmatrix} \mathbf{P}_1^1 \\ \mathbf{P}_2^1 \\ \mathbf{P}_3^1 \end{bmatrix} = \begin{bmatrix} 0,0 \\ 2,3 \\ 4,1 \end{bmatrix}, \quad \theta = \begin{bmatrix} \theta_1^1 \\ \theta_2^1 \\ \theta_3^1 \end{bmatrix} = \begin{bmatrix} 0^\circ \\ -30^\circ \\ -5^\circ \end{bmatrix}. \tag{54}$$

The prescribed configurations of the three-plate-six-bar (3P6B) structure are listed as follows:

$$\mathbf{P} = \begin{bmatrix} \mathbf{P}_1^2 \\ \mathbf{P}_2^2 \\ \mathbf{P}_3^2 \\ \mathbf{P}_1^3 \\ \mathbf{P}_2^3 \\ \mathbf{P}_3^3 \end{bmatrix} = \begin{bmatrix} -2,-1 \\ -1.5,2 \\ 3,0 \\ -3,1 \\ 1.5,2 \\ 1.5,-2 \end{bmatrix}, \quad \theta = \begin{bmatrix} \theta_1^2 \\ \theta_2^2 \\ \theta_3^2 \\ \theta_1^3 \\ \theta_2^3 \\ \theta_3^3 \end{bmatrix} = \begin{bmatrix} 0^\circ \\ 30^\circ \\ 60^\circ \\ 0^\circ \\ 30^\circ \\ 15^\circ \end{bmatrix}. \tag{55}$$

The prescribed configurations of the real-2D two-plate-three-bar structure are listed as follows:

$$\mathbf{P} = \begin{bmatrix} \mathbf{P}_1^1 \\ \mathbf{P}_2^1 \\ \mathbf{P}_3^1 \end{bmatrix} = \begin{bmatrix} -2,-1 \\ -1.5,-2 \\ 3,0 \end{bmatrix}, \quad \theta = \begin{bmatrix} \theta_1^1 \\ \theta_2^1 \\ \theta_3^1 \end{bmatrix} = \begin{bmatrix} 0^\circ \\ 30^\circ \\ 60^\circ \end{bmatrix}. \tag{56}$$

For the quadra-stable structure with local stiffness design, one plate is fixed at the ground (0, 0), the coordinates of the reference point of another plate are prescribed smaller gradually as follows:

$$\mathbf{P} = \begin{bmatrix} \mathbf{P}_1^1 \\ \mathbf{P}_2^1 \\ \mathbf{P}_3^1 \\ \mathbf{P}_4^1 \end{bmatrix} = \begin{bmatrix} 0,-19 \\ 0,-16 \\ 0,-13 \\ 0,-11 \end{bmatrix}, \quad \theta = \begin{bmatrix} \theta_1^1 \\ \theta_2^1 \\ \theta_3^1 \\ \theta_4^1 \end{bmatrix} = \begin{bmatrix} 0^\circ \\ 30^\circ \\ 60^\circ \\ 90^\circ \end{bmatrix}. \tag{57}$$

11.2 Dimensionality reduction of the data

To plot the high dimensional minimum energy path (MEP) in three dimensions, the dimensionality reduction of the data is conducted by the Principal Component Analysis (PCA) method, and MATLAB's built-in function "pca" is used. This section takes the 2P4B structure as an example to illustrate the transformation between the relevant degrees of freedom. The original data of the MEP from Config. 1 to Config. 2 of the 2P4B structure as shown in the black curve in Fig. 4a is written as Eq. (58).

$$\mathbf{CFG} = \begin{bmatrix} 2.25 & -0.02 & 2.74 & 0.08 & 3.00 & 0.51 & 2.86 & 0.99 & 0.39 & -0.92 & 0.81 & -0.58 & 1.00 & 0.06 & 0.84 & 0.55 & 0 & 0 \\ 2.25 & -0.02 & 2.74 & 0.08 & 3.00 & 0.51 & 2.86 & 0.99 & 0.36 & -0.84 & 0.79 & -0.51 & 0.98 & 0.13 & 0.82 & 0.62 & -0.02 & 0.08 \\ \vdots & \vdots & \vdots & \vdots & \vdots & \vdots & \vdots & \vdots & \vdots & \vdots & \vdots & \vdots & \vdots & \vdots & \vdots & \vdots & \vdots & \vdots \\ 2.25 & -0.02 & 2.74 & 0.08 & 3.00 & 0.51 & 2.86 & 0.99 & 1.88 & 2.01 & 2.42 & 2.09 & 2.90 & 2.56 & 3.00 & 3.06 & 2 & 3 \end{bmatrix} \tag{58}$$

where **CFG** is a 52×18 matrix, in which one row represents one configuration on the MEP and the number of rows denotes how many discrete configurations form the continuous MEP. The number of elements in one row illustrates the DOF of the structure.

Then input the matrix **CFG** as the feature matrix to the MATLAB's built-in function ”pca”, a coefficient matrix **ce** that records the coefficients for each principal component (PC) and a matrix **sc** that records the coordinates of the feature matrix in the PC space are obtained.

$$\mathbf{ce} = \begin{bmatrix}
0.00 & 0.00 & 0.00 & 0.00 & 0.00 & 0.00 & 0.00 & 0.00 & 0.00 & 0.00 & 0.75 & -0.25 & 0.58 & -0.18 & -0.01 & 0.00 & 0.00 & 0.00 \\
0.00 & 0.00 & 0.00 & 0.00 & 0.00 & 0.00 & 0.00 & 0.00 & 0.00 & 0.00 & 0.00 & 0.91 & 0.30 & -0.27 & -0.03 & 0.00 & 0.00 & 0.00 \\
0.00 & 0.00 & 0.00 & 0.00 & 0.00 & 0.00 & 0.00 & 0.00 & 0.00 & 0.00 & 0.38 & 0.30 & -0.10 & 0.86 & 0.12 & 0.00 & 0.00 & 0.00 \\
0.00 & 0.00 & 0.00 & 0.00 & 0.00 & 0.00 & 0.00 & 0.00 & 0.00 & 0.00 & 0.00 & 0.00 & -0.03 & -0.14 & 0.99 & 0.01 & 0.00 & 0.00 \\
0.00 & 0.00 & 0.00 & 0.00 & 0.00 & 0.00 & 0.00 & 0.00 & 0.00 & 0.00 & -0.38 & -0.10 & 0.53 & 0.26 & 0.04 & 0.71 & -0.04 & 0.00 \\
0.00 & 0.00 & 0.00 & 0.00 & 0.00 & 0.00 & 0.00 & 0.00 & 0.00 & 0.00 & 0.00 & 0.00 & 0.00 & 0.00 & 0.00 & 0.05 & 0.95 & 0.32 \\
0.00 & 0.00 & 0.00 & 0.00 & 0.00 & 0.00 & 0.00 & 0.00 & 0.00 & 0.00 & 0.38 & 0.10 & -0.53 & -0.25 & -0.06 & 0.71 & -0.04 & 0.00 \\
0.00 & 0.00 & 0.00 & 0.00 & 0.00 & 0.00 & 0.00 & 0.00 & 0.00 & 0.00 & 0.00 & 0.00 & 0.00 & 0.00 & 0.00 & -0.01 & -0.32 & 0.95 \\
0.21 & 0.38 & 0.50 & 0.25 & -0.11 & -0.20 & 0.65 & -0.01 & -0.15 & -0.03 & 0.00 & 0.00 & 0.00 & 0.00 & 0.00 & 0.00 & 0.00 & 0.00 \\
0.40 & -0.24 & -0.25 & 0.50 & -0.18 & -0.03 & 0.03 & 0.54 & 0.31 & -0.23 & 0.00 & 0.00 & 0.00 & 0.00 & 0.00 & 0.00 & 0.00 & 0.00 \\
0.22 & 0.37 & 0.33 & -0.03 & 0.00 & -0.04 & -0.37 & -0.23 & 0.71 & -0.08 & 0.00 & 0.00 & 0.00 & 0.00 & 0.00 & 0.00 & 0.00 & 0.00 \\
0.36 & -0.24 & 0.08 & 0.30 & 0.57 & -0.24 & -0.13 & -0.16 & -0.09 & 0.53 & 0.00 & 0.00 & 0.00 & 0.00 & 0.00 & 0.00 & 0.00 & 0.00 \\
0.26 & 0.36 & -0.08 & -0.29 & -0.35 & -0.01 & -0.19 & 0.42 & -0.14 & 0.60 & 0.00 & 0.00 & 0.00 & 0.00 & 0.00 & 0.00 & 0.00 & 0.00 \\
0.34 & -0.25 & 0.33 & -0.11 & -0.29 & -0.29 & -0.44 & -0.08 & -0.45 & -0.36 & 0.00 & 0.00 & 0.00 & 0.00 & 0.00 & 0.00 & 0.00 & 0.00 \\
0.29 & 0.37 & -0.42 & -0.35 & 0.45 & -0.34 & 0.08 & 0.10 & -0.09 & -0.37 & 0.00 & 0.00 & 0.00 & 0.00 & 0.00 & 0.00 & 0.00 & 0.00 \\
0.34 & -0.26 & 0.34 & -0.43 & 0.28 & 0.58 & 0.19 & 0.24 & 0.05 & -0.08 & 0.00 & 0.00 & 0.00 & 0.00 & 0.00 & 0.00 & 0.00 & 0.00 \\
0.27 & 0.38 & -0.21 & 0.37 & 0.01 & 0.60 & -0.18 & -0.29 & -0.33 & -0.11 & 0.00 & 0.00 & 0.00 & 0.00 & 0.00 & 0.00 & 0.00 & 0.00 \\
0.40 & -0.25 & -0.35 & -0.23 & -0.38 & -0.03 & 0.34 & -0.54 & 0.18 & 0.13 & 0.00 & 0.00 & 0.00 & 0.00 & 0.00 & 0.00 & 0.00 & 0.00
\end{bmatrix} \tag{59}$$

where **ce** is an 18×18 matrix, in which each column contains coefficients for one principal component and the columns are in descending order of component variance. The matrix **sc** is also a 52×18 matrix, where each row corresponds to a row in the original data matrix **CFG** and each column corresponds to a principal component. Therefore, to plot the Path 1-2 (i.e. the MEP from Config. 1 to Config. 2) in three dimensions as shown in Fig. 4a, the first two columns are extracted from the matrix **sc** as the x-axis and y-axis of the principal component space as Eq. (60), the z-axis is filled with the values of

the physical energy corresponding to 52 configurations on the MEP.

$$
\mathbf{sc}(:,1:2)' = \begin{bmatrix}
-3.67 & 1.12 \\
-3.56 & 1.00 \\
-3.45 & 0.87 \\
-3.33 & 0.75 \\
-3.21 & 0.64 \\
-3.09 & 0.53 \\
-2.96 & 0.42 \\
-2.83 & 0.32 \\
-2.69 & 0.23 \\
-2.56 & 0.14 \\
-2.42 & 0.05 \\
-2.27 & -0.03 \\
-2.13 & -0.11 \\
-1.98 & -0.18 \\
-1.83 & -0.24 \\
-1.68 & -0.30 \\
-1.53 & -0.36 \\
-1.37 & -0.41 \\
-1.21 & -0.45 \\
-1.05 & -0.49 \\
-0.90 & -0.53 \\
-0.73 & -0.55 \\
-0.57 & -0.58 \\
-0.41 & -0.59 \\
-0.25 & -0.61 \\
-0.09 & -0.61 \\
0.08 & -0.61 \\
0.24 & -0.61 \\
0.40 & -0.60 \\
0.56 & -0.58 \\
0.72 & -0.56 \\
0.88 & -0.53 \\
1.04 & -0.50 \\
1.20 & -0.46 \\
1.36 & -0.42 \\
1.52 & -0.37 \\
1.67 & -0.31 \\
1.83 & -0.25 \\
1.98 & -0.18 \\
2.13 & -0.11 \\
2.27 & -0.04 \\
2.42 & 0.04 \\
2.56 & 0.13 \\
2.70 & 0.22 \\
2.84 & 0.32 \\
2.97 & 0.42 \\
3.10 & 0.53 \\
3.23 & 0.64 \\
3.35 & 0.76 \\
3.47 & 0.88 \\
3.58 & 1.00 \\
3.69 & 1.13
\end{bmatrix}, \quad
\mathbf{E_{phy}} = \begin{bmatrix}
0.00E+00 \\
2.13E-05 \\
8.11E-05 \\
1.74E-04 \\
2.95E-04 \\
4.40E-04 \\
6.05E-04 \\
7.84E-04 \\
9.78E-04 \\
1.17E-03 \\
1.37E-03 \\
1.57E-03 \\
1.77E-03 \\
1.97E-03 \\
2.16E-03 \\
2.34E-03 \\
2.52E-03 \\
2.68E-03 \\
2.83E-03 \\
2.96E-03 \\
3.08E-03 \\
3.18E-03 \\
3.26E-03 \\
3.33E-03 \\
3.38E-03 \\
3.40E-03 \\
3.41E-03 \\
3.40E-03 \\
3.37E-03 \\
3.31E-03 \\
3.24E-03 \\
3.16E-03 \\
3.05E-03 \\
2.92E-03 \\
2.78E-03 \\
2.63E-03 \\
2.46E-03 \\
2.28E-03 \\
2.09E-03 \\
1.89E-03 \\
1.69E-03 \\
1.48E-03 \\
1.27E-03 \\
1.06E-03 \\
8.58E-04 \\
6.68E-04 \\
4.92E-04 \\
3.34E-04 \\
2.01E-04 \\
9.82E-05 \\
3.06E-05 \\
0.00E+00
\end{bmatrix} \tag{60}
$$

11.3 Additional stable states demonstration for the three-plate-six-bar structure

The topology of the three-plate-six-bar structure is more complex, so there are more additional stable states different from prescribed configurations. The physical model is randomly actuated to other directions different from the designed and recorded photographs of additional stable states as Supplementary Fig. 21. Therefore, it is necessary to employ the NEB method to identify the minimum energy path and design the actuation guiding the structure to reconfigure along the path.

11.4 Step-by-step design procedure of the multi-stable 2P4B structure

The design procedure of a simple multi-stable 2P4B structure is demonstrated step-by-step. In two dimensions, bodies are constrained in a plane to be plates. So the structure consists of six plates, nb=6, as shown in Supplementary Fig. 22a. This structure was designed with three stable configurations and a given topology. The only design variables are the coordinates of all joints A. All computations were carried out with MATLAB(2020).

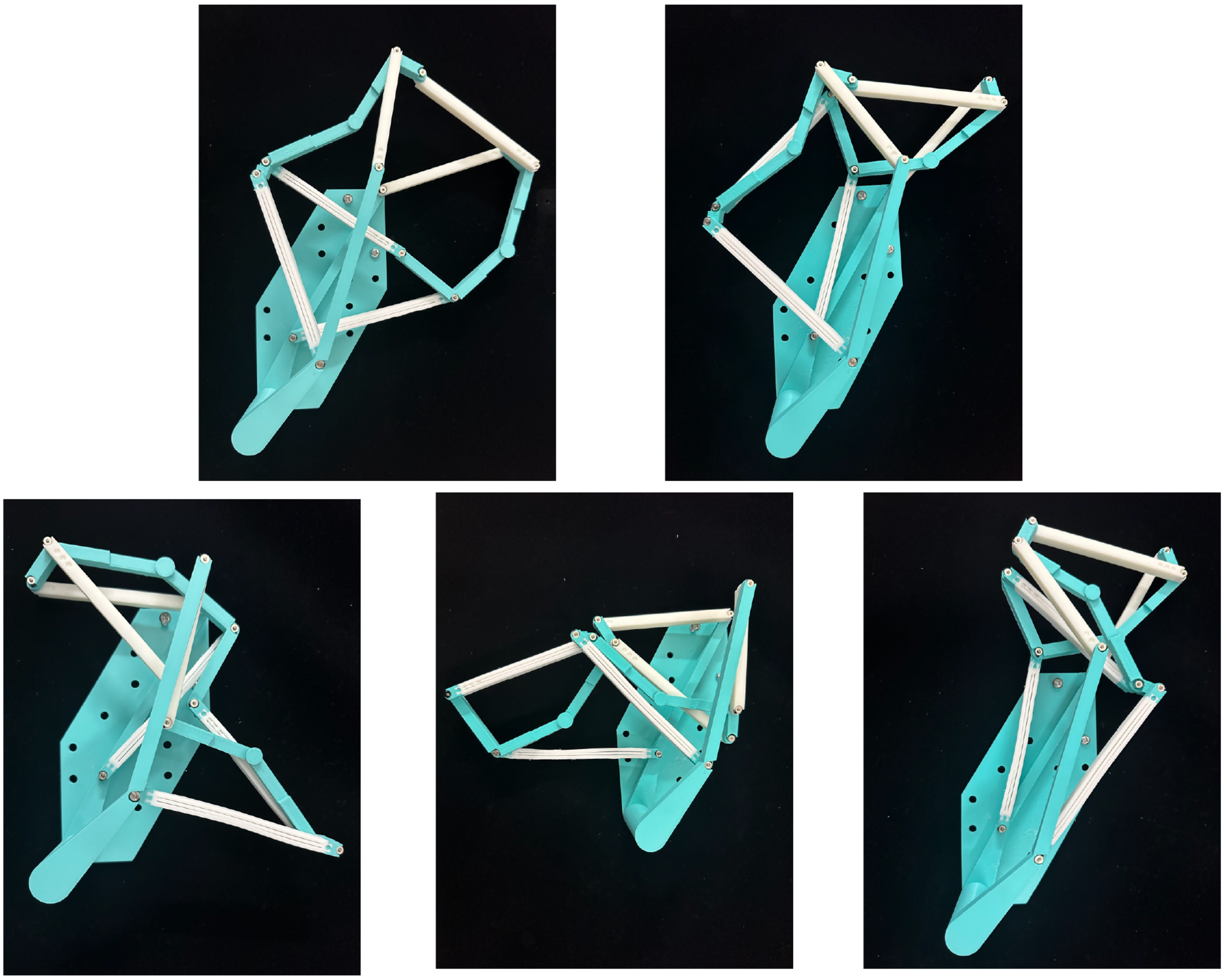

Supplementary Fig. 21. **The demonstration of the additional stable state of the three-plate-six-bar multi-stable structure.** Intuitively, we played with the physical model and found five additional stable states that are different from the three prescribed target configurations. Two additional stable states in the first row are predicted through the NEB method.

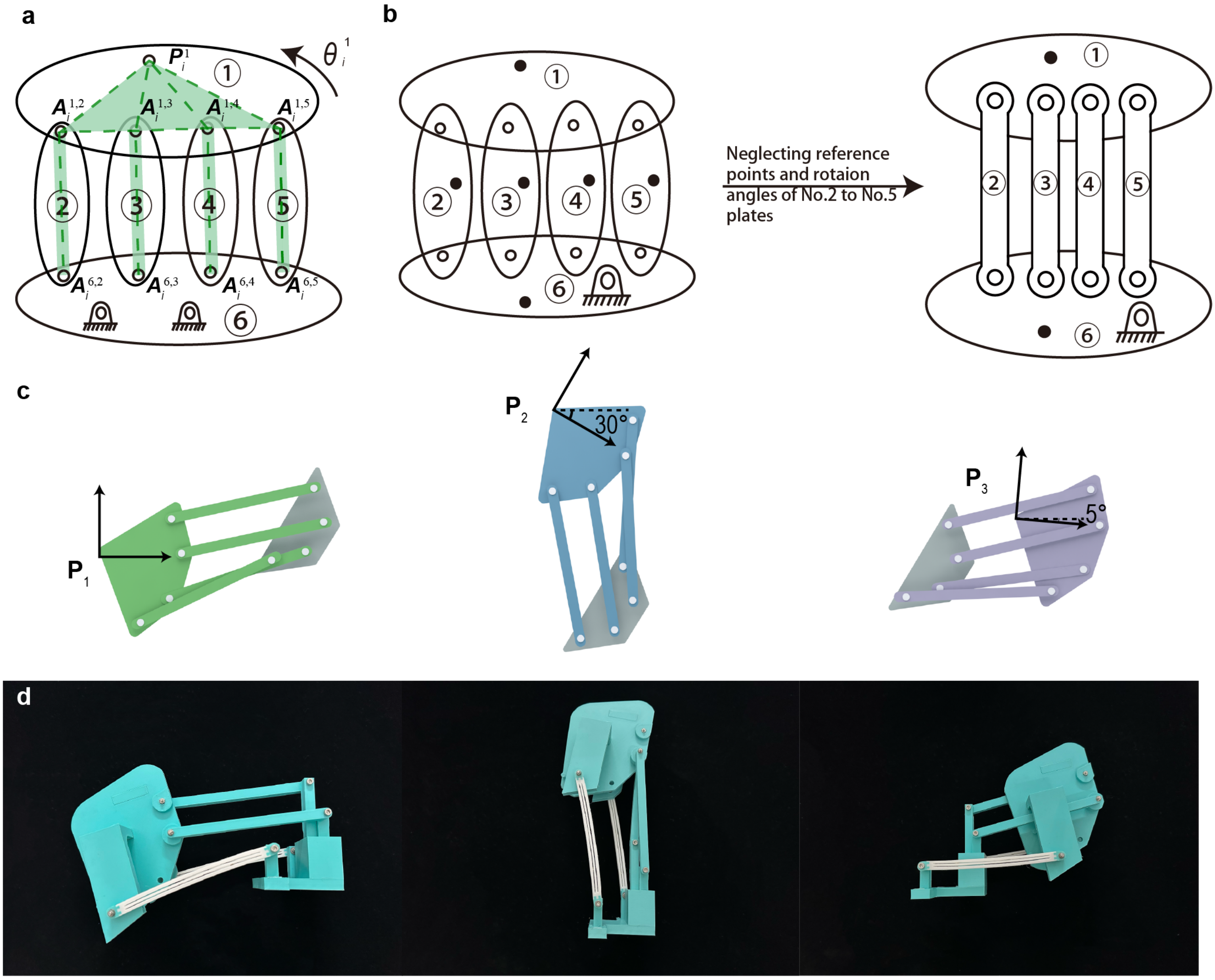

Supplementary Fig. 22 **The design procedure of the multi-stable 2P4B structure. a** Notations of the variables on the structure. **b** Plates 2-5 can be simplified to bars when only prescribed the target configurations of plate 1 and plate 6 and that of plates 2-5 are out of interest. **c** The result of the constrained optimiztion of the multi-compatibility structure design. **d** The corresponding physical model.

11.4.1 Step A: Prescribe information about each target configuration

Assume only plate 1 and plate 6 are of interest, which means only reference points and rotation angles of these two plates are prescribed and others are set as variables. So neglecting reference points of plate 2 to plate 5, these plates are simplified as bars, as shown in Supplementary Fig. 22b. Then that two plates are connected by four bars is the topology in this example. Only the poses (reference points and rotation angles) of these plates are of interest, one plate was fixed at ground and its reference points at three target configurations were set as [0,0], its rotation angles were set as 0. The reference points of the other plate were prescribed at three target configurations, nc=3, shown in Eq. (61),(62).

$$\mathbf{P} = \begin{bmatrix} \mathbf{P}_1^1 \\ \mathbf{P}_2^1 \\ \mathbf{P}_3^1 \\ \mathbf{P}_1^6 \\ \mathbf{P}_2^6 \\ \mathbf{P}_3^6 \end{bmatrix} = \begin{bmatrix} 0,0 \\ 2,3 \\ 4,1 \\ 0,0 \\ 0,0 \\ 0,0 \end{bmatrix} \tag{61}$$

$$\theta = \begin{bmatrix} \theta_1^1 \\ \theta_2^1 \\ \theta_3^1 \\ \theta_1^6 \\ \theta_2^6 \\ \theta_3^6 \end{bmatrix} = \begin{bmatrix} 0\,^\circ \\ 30\,^\circ \\ 5\,^\circ \\ 0\,^\circ \\ 0\,^\circ \\ 0\,^\circ \end{bmatrix} \tag{62}$$

11.4.2 Step B: Conduct constrained optimization on the geometric configuration

For two dimension, the rotation matrix should be:

$$Rot(\bullet) = \begin{bmatrix} \cos\theta & -\sin\theta \\ \sin\theta & \cos\theta \end{bmatrix} \tag{63}$$

The rotation equations of these six components from configuration 1 to configuration 2 and 3 could be written. The target configurations of body 1 are prescribed as Eq. (61)-(62), the rotation equations of it are:

$$\begin{aligned} \mathbf{A}_2^{1,p} - \begin{bmatrix} 2 \\ 3 \end{bmatrix} &= \begin{bmatrix} 0.8660 & -0.5000 \\ 0.5000 & 0.8660 \end{bmatrix} \cdot (\mathbf{A}_1^{1,p} - \begin{bmatrix} 0 \\ 0 \end{bmatrix}) \\ \mathbf{A}_3^{1,p} - \begin{bmatrix} 4 \\ 1 \end{bmatrix} &= \begin{bmatrix} 0.9962 & -0.0872 \\ 0.0872 & 0.9962 \end{bmatrix} \cdot (\mathbf{A}_1^{1,p} - \begin{bmatrix} 0 \\ 0 \end{bmatrix}) \end{aligned} \tag{64}$$

where p equals to 2-5 because body 1 is connected with body 2, 3, 4, 5. Then all A points at other configurations could be expressed by all A points at configuration 1 and only A points at configuration 1 are regarded as variables. Above equations could be transformed into following forms:

$$\begin{aligned} \mathbf{A}_2^{1,p} &= \begin{bmatrix} 2 \\ 3 \end{bmatrix} + \begin{bmatrix} 0.8660 & -0.5000 \\ 0.5000 & 0.8660 \end{bmatrix} \cdot (\mathbf{A}_1^{1,p} - \begin{bmatrix} 0 \\ 0 \end{bmatrix}) \\ \mathbf{A}_3^{1,p} &= \begin{bmatrix} 4 \\ 1 \end{bmatrix} + \begin{bmatrix} 0.9962 & -0.0872 \\ 0.0872 & 0.9962 \end{bmatrix} \cdot (\mathbf{A}_1^{1,p} - \begin{bmatrix} 0 \\ 0 \end{bmatrix}) \end{aligned} \tag{65}$$

Considering the poses of plate 2 to plate 5 are of no interest, the reference points and rotation angles of these plates are regarded as variables, their rotation equations could be written as follows:

$$\mathbf{A}_i^{p,q} - \mathbf{P}_i^p = \begin{bmatrix} \cos\theta_i^p & -\sin\theta_i^p \\ \sin\theta_i^p & \cos\theta_i^p \end{bmatrix} \cdot (\mathbf{A}_1^{p,q} - \mathbf{P}_1^p) \tag{66}$$

where p=2,..,5, i=2, 3, because components 2-5 are all connected between plate 1 and plate 6, so q equals to 1 and 6. P_i^p, θ_i^p and $A_1^{p,q}$ are variables and $A_i^{p,q}$ could be expressed by above variables as follows:

$$\mathbf{A}_i^{p,q} = \mathbf{P}_i^p + \begin{bmatrix} \cos\theta_i^p & -\sin\theta_i^p \\ \sin\theta_i^p & \cos\theta_i^p \end{bmatrix} \cdot (\mathbf{A}_1^{p,q} - \mathbf{P}_1^p) \tag{67}$$

Because the plate 6 was fixed at the ground, there is no need to write its rotation equations.

Above rotation equations ensure the size of all components unchanged at three target configurations. The condition about all joints as Eq. (11) were written as equality constraints:

$$Ceq_{cpt}(\mathbf{A}) = \left\{ \begin{matrix} |\mathbf{A}_i^{1,q1} - \mathbf{A}_i^{q1,1}| = 0\ , & q1 = 2,3,4,5 \\ |\mathbf{A}_i^{6,q6} - \mathbf{A}_i^{q6,6}| = 0\ , & q6 = 2,3,4,5 \end{matrix} \ , \quad i = 1,2,3 \right\} \tag{68}$$

Except for fundamental two conditions, two categories of inequality constraints were added to limit the size of these components, one category of inequality constraints describes the distance between the reference point and the joint points, presented as follows:

$$C_{size}(\mathbf{A}) = \left\{ \begin{matrix} 1 \le |\mathbf{P}_1^1 - \mathbf{A}_1^{1,q1}| \le 10\ , q1 = 2,3,4,5 \\ 1 \le |\mathbf{P}_1^6 - \mathbf{A}_1^{6,q6}| \le 10\ , q6 = 2,3,4,5 \end{matrix} \right\} \tag{69}$$

Another category of inequality constraints limits the distance among joints, the lower bound setting prevents these joints overlapping, the upper bound setting limits the size of bodies, shown as follows:

$$C_{size}(\mathbf{A}) = \left\{ \begin{matrix} 1 \le |\mathbf{A}_1^{p,2} - \mathbf{A}_1^{p,3}| \le 10 \\ 1 \le |\mathbf{A}_1^{p,2} - \mathbf{A}_1^{p,4}| \le 10 \\ 1 \le |\mathbf{A}_1^{p,2} - \mathbf{A}_1^{p,5}| \le 10 \\ 1 \le |\mathbf{A}_1^{p,3} - \mathbf{A}_1^{p,4}| \le 10 \\ 1 \le |\mathbf{A}_1^{p,3} - \mathbf{A}_1^{p,5}| \le 10 \\ 1 \le |\mathbf{A}_1^{p,4} - \mathbf{A}_1^{p,5}| \le 10 \end{matrix} , p = 1,6 \right\} \tag{70}$$

Plate 2 to 5 were simplified as bars without reference points, so only the second term in Eq. (15) is considered for plate 2 to 5:

$$\begin{aligned} E(\mathbf{A}) = &\sum_{p=2,3,4,5} |\mathbf{A}_1^{p,1} - \mathbf{A}_1^{p,6}| + \sum_{q1=2,3,4,5} |\mathbf{P}_1^1 - \mathbf{A}_1^{1,q1}| + \sum_{q6=2,3,4,5} |\mathbf{P}_1^6 - \mathbf{A}_1^{6,q6}| \\ &+ \sum_{p=1,6} (|\mathbf{A}_1^{p,2} - \mathbf{A}_1^{p,3}| + |\mathbf{A}_1^{p,2} - \mathbf{A}_1^{p,4}| + |\mathbf{A}_1^{p,2} - \mathbf{A}_1^{p,5}| + |\mathbf{A}_1^{p,3} - \mathbf{A}_1^{p,4}| \\ &+ |\mathbf{A}_1^{p,3} - \mathbf{A}_1^{p,5}| + |\mathbf{A}_1^{p,4} - \mathbf{A}_1^{p,5}|) \end{aligned} \tag{71}$$

11.4.3 Solutions

The options of the nonlinear optimization solver "fmincon" were set as default. The results of the optimization are shown in Tab. 1 and Fig. 22c. Because plate 6 was fixed at the ground, the coordinates of joints were the same at different target configurations. In Fig. 22c, the grey part means component 6 fixed at the ground while the other three plates with colors denote component 1 at three target configurations, and bars between two plates represent components 2 to 5.

Config.(i)	$A_i^{1,2}$	$A_i^{1,3}$	$A_i^{1,4}$	$A_i^{1,5}$
1	[0.3949,-0.9187]	[0.8144,-0.5803]	[0.9980,0.0635]	[0.8359,0.5488]
2	[1.8826,2.0069]	[2.4152,2.0903]	[2.8960,2.5526]	[2.9984,3.0573]
3	[4.3133,0.0504]	[4.7607,0.3509]	[4.9997,0.9763]	[4.8806,1.4739]
Config.(i)	$A_i^{6,2}$	$A_i^{6,3}$	$A_i^{6,4}$	$A_i^{6,5}$
1	[2.2522,-0.0221]	[2.7418,0.0795]	[3.0019,0.5065]	[2.8638,0.9870]
2	[2.2522,-0.0221]	[2.7418,0.0795]	[3.0019,0.5065]	[2.8638,0.9870]
3	[2.2522,-0.0221]	[2.7418,0.0795]	[3.0019,0.5065]	[2.8638,0.9870]

Table 1: 2D example solution from MATLAB

References

[1] ET Filipov et al. "Bar and hinge models for scalable analysis of origami". In: *International Journal of Solids and Structures* 124 (2017), pp. 26–45.

[2] Sergio Pellegrino and Christopher Reuben Calladine. "Matrix analysis of statically and kinematically indeterminate frameworks". In: *International Journal of Solids and Structures* 22.4 (1986), pp. 409–428.

[3] Yang Li and Sergio Pellegrino. "A Theory for the Design of Multi-Stable Morphing Structures". In: *Journal of the Mechanics and Physics of Solids* 136 (2020). ISSN: 00225096. DOI: 10.1016/j.jmps.2019.103772.

[4] Henry C Herbol, James Stevenson, and Paulette Clancy. "Computational implementation of nudged elastic band, rigid rotation, and corresponding force optimization". In: *Journal of Chemical Theory and Computation* 13.7 (2017), pp. 3250–3259.

[5] Hao Zhou et al. "Low energy fold paths in multistable origami structures". In: *International Journal of Solids and Structures* (2023), p. 112125.

[6] Jingyi Yang et al. "Folding and deploying identical thick panels with spring-loaded hinges". In: *Extreme Mechanics Letters* 52 (2022), p. 101637.

[7] Hiroaki FUNABASHI and Kiyoshi OGAWA. "On the design of planar mechanisms with consideration of interferences of moving links". In: *Bulletin of JSME* 27.224 (1984), pp. 341–347.

[8] Ran Zhang, Thomas Auzinger, and Bernd Bickel. "Computational design of planar multistable compliant structures". In: *ACM Transactions on Graphics (TOG)* 40.5 (2021), pp. 1–16.

[9] Peter Wriggers. *Nonlinear finite element methods*. Springer Science & Business Media, 2008.

[10] Haitao Ye et al. "Multimaterial 3D printed self-locking thick-panel origami metamaterials". In: *Nature Communications* 14.1 (2023), p. 1607.
