# Supplementary material for: Easy-to-actuate multi-compatible truss structures with prescribed reconfiguration
Source: Nat Commun. 2024 Jun 7;15:4886. doi: 10.1038/s41467-024-49210-3 (PMC11161661; doi:10.1038/s41467-024-49210-3)
Supplement: Supplementary file 3 — Description of Additional Supplementary Files [file 41467_2024_49210_MOESM3_ESM.pdf]

Description of Additional Supplementary Files

Supplementary Movie 1: the design procedure of the 2plate-4bar tri-stable structure and the reconfiguration of the prototype actuated by the calculated actuator

Supplementary Movie 2: the design procedure of the 3plate-6bar tri-stable structure, the reconfiguration of the prototype actuated by the calculated actuator, and the demonstration of additional stable states

Supplementary Movie 3: the reconfiguration of an interference-free tri-stable 2D unit cell and that of its assembly

Supplementary Movie 4: the stiffness design of a quadra-stable unit cell and its assembly, the reconfiguration of the prototypes

Supplementary Movie 5: apply a quadra-stable structure to the design of a variable stiffness gripper, and use only one actuator to operate the gripper in the two working modes as well as switch between the two modes to grasp a tofu

Supplementary Code 1: the step-by-step design procedure for the simplest example (the 2P4B structure).

Supplementary Code 2: a general design toolbox for customizing the multi-stable structure.
